# Modeling and Simulation of Optimal Resource Management during the Diurnal Cycle in *Emiliania huxleyi* by Genome-Scale Reconstruction and an Extended Flux Balance Analysis Approach

**DOI:** 10.3390/metabo5040659

**Published:** 2015-10-28

**Authors:** David Knies, Philipp Wittmüß, Sebastian Appel, Oliver Sawodny, Michael Ederer, Ronny Feuer

**Affiliations:** Institute for System Dynamics, University of Stuttgart, Waldburgstrasse 17/19, Stuttgart 70563, Germany; E-Mails: david.knies@isys.uni-stuttgart.de (D.K.); philipp.wittmuess@isys.uni-stuttgart.de (P.W.); Sebastianappel@hotmail.com (S.A.); oliver.sawodny@isys.uni-stuttgart.de (O.S.); michael.ederer@isys.uni-stuttgart.de (M.E.)

**Keywords:** *Emiliania huxleyi*, genome-scale model, flux balance analysis, diurnal cycle

## Abstract

The coccolithophorid unicellular alga *Emiliania huxleyi* is known to form large blooms, which have a strong effect on the marine carbon cycle. As a photosynthetic organism, it is subjected to a circadian rhythm due to the changing light conditions throughout the day. For a better understanding of the metabolic processes under these periodically-changing environmental conditions, a genome-scale model based on a genome reconstruction of the *E. huxleyi* strain CCMP 1516 was created. It comprises 410 reactions and 363 metabolites. Biomass composition is variable based on the differentiation into functional biomass components and storage metabolites. The model is analyzed with a flux balance analysis approach called diurnal flux balance analysis (diuFBA) that was designed for organisms with a circadian rhythm. It allows storage metabolites to accumulate or be consumed over the diurnal cycle, while keeping the structure of a classical FBA problem. A feature of this approach is that the production and consumption of storage metabolites is not defined externally via the biomass composition, but the result of optimal resource management adapted to the diurnally-changing environmental conditions. The model in combination with this approach is able to simulate the variable biomass composition during the diurnal cycle in proximity to literature data.

## 1. Introduction

*Emiliania huxleyi* is a species of unicellular coccolithophorid algae. As a coccolithophore, it forms highly-structured calcium carbonate plates (coccoliths). *E. huxleyi* blooms can be seen as a major carbon sink in the global carbon cycle [1]. They have an influence on regional and global climate by consuming greenhouse gases and the produced coccoliths changing the albedo of the ocean [2]. This biomineralization process also makes them interesting targets for biotechnology, resulting in the need for detailed metabolic modeling. A first step in this direction was taken by the genome analysis performed by Read *et al.* [3]. This study showed a high genetic variability of different strains of *E. huxleyi* sampled all over the world. The genome of strain CCMP 1516 was annotated as a reference genome. To make this data usable for metabolic engineering techniques in the form of constraint-based analysis, the genome-scale metabolic network needs to be reconstructed.

Genome-scale reconstructions of many organisms are available today and are still being updated. For example, models of *Escherichia coli* [4,5], *Saccharomyces cerevisiae* [6], *Arabidopsis thaliana* [7] and *Chlamydomonas reinhardtii* [8] are available, but no reconstruction for *E. huxleyi* is available, yet.

Constraint-based analysis of metabolic networks with methods, like flux balance analysis (FBA), phenotypic phase plane (PhPP) analysis and dynamic FBA, is widely used in systems biology [9,10,11,12,13]. These methods use stoichiometric information of reactions, thermodynamic restrictions on reaction directions and additional constraints. Assuming a quasi-steady state of internal metabolites, convex optimization techniques can be applied to compute optimal flux distributions for the metabolic network with regard to the environmental conditions. In the biological system, the flux distribution is controlled by a complex network of regulators and transcription factors, allowing the cell to reroute its resources to adjust to the current environmental conditions.

An important task while setting up FBA problems is to define an appropriate objective function that results in realistic flux distributions. Typically, objectives, such as “maximize biomass production”, “maximize ATP production” or “minimize substrate uptake”, are chosen [9]. These approaches have been successfully applied to organisms that are confronted with non-periodically change and substrate-limited environmental conditions [6,14]. Since *E. huxleyi* is adapted to diurnally-changing environmental conditions with alternating resource availability, an approach considering the diurnal change is required. Therefore, using the organism’s currently-available resources as efficiently as possible towards biomass production might not be sufficient. A more complex optimization goal has to be stated for these organisms, namely “Maximize biomass production and produce enough storage metabolites to survive a well-defined starvation period”.

In contrast, previous publications applying the classical FBA to plants and algae fix the proportion of storage metabolites in the biomass function for the light metabolism [7,8]. As a result, the amount of storage metabolites produced cannot be determined via optimization, but is rather defined by measurement data used when formulating the biomass function. This also means that the production of storage metabolites is coupled to growth, which are two processes that are competing for the cell’s resources. Another flaw of classical FBA approaches on photosynthetic organisms is that for these, a steady state or a constant biomass composition cannot be assumed if all metabolites, including storage metabolites, are considered.

Many extensions of the FBA approach were developed, for example the dynamic FBA, which allows the dynamic simulation of biological processes by balancing the external metabolites via uptake and excretion kinetics [13]. The dynamic FBA dynamic optimization approach (dFBA DOA) allows the implementation of more complex optimality criteria, but can result in a non-convex optimization problem [13], which is harder to solve than a convex one.

The approach presented in this work, a diurnal flux balance analysis (diuFBA), is similar to the integrated FBA proposed by Cheung *et al.*, who duplicated the fluxes for a stoichiometric model of *Arabidopsis thaliana* to simulate day and night metabolism in a single optimization problem [15].

This is related to the dFBA DOA, but contains some simplifying assumptions. The approach is based on dividing the diurnal cycle into periods of quasi-steady-state environmental conditions, namely a light and a dark period, and to solve an optimization problem over a complete diurnal cycle. The time steps are merged into a single stoichiometric matrix and linked by explicit Euler integration steps, making it possible to formulate a convex optimization problem that is similar to a classical FBA problem.

In this study, we introduce a genome-scale reconstruction of *E. huxleyi* and apply the diuFBA formalism. We demonstrate the diuFBA approach on an example model. The diuFBA is then applied to the genome-scale reconstruction to simulate a diurnal cycle, with the focus on the biomass composition at the transition from day to night.

## 2. Experimental Section

### 2.1. Software

Modeling was performed using the COBRA Toolbox 2.0 [16] with MATLAB 2013a 64 bit (The MathWorks, Inc., Natick, MA, USA) and rBioNet [17] optimization using the COBRA Toolbox 2.0 and Gurobi 5.6.0 [18]. A custom MATLAB script (see Supplementary Material S2) was used to generate the extended stoichiometric matrix from a COBRA Toolbox model structure.

### 2.2. Genome-Scale Model of E. huxleyi

Recently, the *E. huxleyi* CCMP 1516 genome was reconstructed and annotated [3]. Based on this reconstruction, the stoichiometric model of the metabolic network of *E. huxleyi* presented in this work was built according to the protocol by Thiele and Palsson [19]. In order to use the modeling approach described later in this section, the biomass function was split up into functional biomass and storage metabolites. The term functional biomass comprises elements of the biomass that are directly necessary for the viability of the cell, such as nucleic acids, proteins, structural lipids and polysaccharides, but excludes components with a long-term benefit, such as polysaccharides and lipids used as storage metabolites. We named the model iEH410 for the 410 reactions it contains.

### 2.3. diuFBA

**Figure 1:**
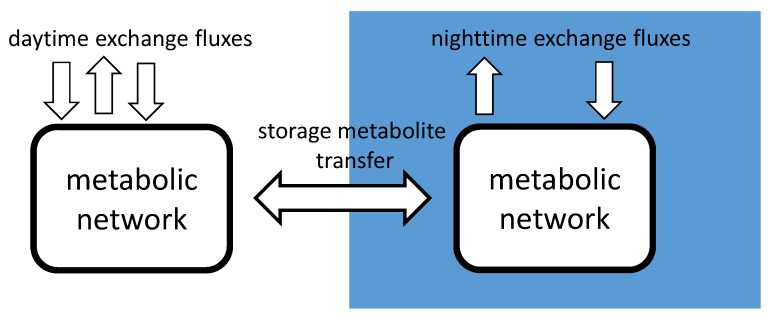
Schematic of the extended flux balance analysis (FBA) approach: the same metabolic network is simulated for both day and nighttime, while storage metabolites are used to transfer resources between the two states.

The general idea of the approach is to have storage metabolites that can be transferred between daytime and nighttime, while each time step is considered as a flux balance problem with varying environmental exchange fluxes. Resources can be exchanged between the two time steps via storage metabolites. This is illustrated in Figure 1.

To depict the behavior of a photosynthetic organism over the course of one day with the diuFBA, we assume that all time steps can be considered to be in a quasi-steady state within themselves. This assumption is valid if we consider the environmental conditions constant and the substrate is abundant within each time step. This is a realistic assumption for cultivation experiments in bioreactors or other monitored environments, but also for beginning blooms, in which no nutrient limitation is present.

We consider a metabolic network with *r* reactions, *s* species, which contain *p* storage metabolites. To describe the connection between day and night metabolism, an extended stoichiometric matrix $\tilde{S}$ is formulated. It consists of the original stoichiometric matrix $S\in R^{s\times r}$ and a transition matrix $T\in R^{2s\times p}$ that allows the transfer of storage metabolites between the two time steps. The transition matrix T is constructed as a set of reactions that links the storage metabolites $c_{\text{storage}}$ between the two time steps:

(1) $$c_{\text{storage,light}}↔c_{\text{storage,dark}}$$

The transfer reactions are modeled as reversible reactions, since the transfer of storage metabolites is possible from day to night, as well as from night to day.

For each time step, one instance of the original matrix S is added to the extended stoichiometric matrix $\tilde{S}$. The two matrices are connected via the transition matrix. This results in the following structure for $\tilde{S}$:

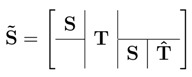
(2)

This definition also includes the shortened transition matrix $\hat{T}\in R^{r\times p}$, which adds an export reaction for storage metabolites. These reactions are used to simulate storage metabolite aggregation beyond the diurnal cycle, such as long-term storage, or biomass growth. This extension results in a stoichiometric matrix $\tilde{S}\in R^{\left(2\;\cdot \;s\right)\times 2\;\cdot \;\left(r+p\right)}$.

As a result, the reaction vector v is extended accordingly to have the dimension $\tilde{v}\in R^{2\;\cdot \;\left(r+p\right)}$:

(3) $$\tilde{v}=\left[\begin{array}{c} \Delta c_{\text{light}}\\ \Delta c_{p}\\ \Delta c_{\text{dark}}\\ \Delta c_{p,ex} \end{array}\right]$$

In this equation, $\Delta c_{p}$ stands for concentration changes between two time steps. These are associated with the transition matrix T, while $\Delta c_{p,ex}$ represents the molar transport out of the system, associated with the shortened transition matrix $\hat{T}$. This concentration change can be used to describe overall biomass growth, or long-term storage effects.

Concentration changes are used due to the time discretization, which results in the reaction vector now containing the integrated stationary fluxes. The integration of a flux *v* (mmol /gDCW /h) (gDCW: grams of dry cell weight) over a time step, e.g., the light phase, results in a change of concentration Δc (mmol /gDCW):

(4) $$\int_{t_{\text{light,start}}}^{t_{\text{light,end}}}v\left(\tau \right)d\tau =v\;\cdot \;\Delta t_{\text{light}}=\Delta c_{\text{light}}$$

where $\Delta t_{\text{light}}$ and $\Delta t_{\text{dark}}$ are the durations of the light and the dark phase, respectively. Therefore, the resulting reaction vector $\Delta c_{\text{light}}$ no longer consists of the change rates of molar amounts, but of the changes of the molar amount in the corresponding time step. This difference is important, as reaction vector components from time steps of differing lengths can no longer be compared directly in the sense of reaction speed. The comparison is still valuable, though, since it shows if a slower built-up reaction in a longer time step is able to satisfy the needs of a faster consumption reaction in a shorter time step. This also reflects that in the new approach, the mass balance for internal storage metabolites does not need to be satisfied in each time step, but in the overall diurnal cycle: even if internal storage metabolites are built up in one time step, the mass balance can still be satisfied if all of the accumulated metabolites are consumed in the other time step. The fluxes for the time steps, which can be compared to classical FBA results, can easily be obtained using the length of day and night phase:

(5) $$v_{\text{light}}=\frac{\Delta c_{\text{light}}}{\Delta t_{\text{light}}};\;v_{\text{dark}}=\frac{\Delta c_{\text{dark}}}{\Delta t_{\text{dark}}}$$

With the extended stoichiometric matrix $\tilde{S}$ and flux vector $\tilde{v}$, the optimization problem can be formulated. It is similar to the classical FBA optimization problem:

(6) $$\begin{array}{c} \max w^{⊤}\tilde{v}\\ s.t.\tilde{S}\tilde{v}=0\\ {\tilde{v}}_{\text{lb}}\leq \tilde{v}\leq {\tilde{v}}_{\text{ub}} \end{array}$$

where w is an objective vector, which is used to weight the fluxes in the flux vector $\tilde{v}$. The vectors ${\tilde{v}}_{\text{lb}}$ and ${\tilde{v}}_{\text{ub}}$ stand for the upper and lower bounds of the flux vector, which describes metabolic rate limitations and reversibility.

### 2.4. Phenotypic Phase Plane Plots

A common tool for the analysis of the metabolic capabilities of a stoichiometric model and its distinct patterns of pathway utilization is phenotypic phase plane (PhPP) analysis [12,20]. For two reactions ${\tilde{v}}_{r1}$ and ${\tilde{v}}_{r2}$, a grid of m×n values on the intervals ${\tilde{v}}_{r1,1},...,{\tilde{v}}_{r1,m}$ and ${\tilde{v}}_{r2,1},...,{\tilde{v}}_{r2,n}$ is created. A diuFBA is performed for each combination of flux values to generate a grid of objective function values:

(7) $$\begin{array}{c} \max w^{⊤}\tilde{v}\left({\tilde{v}}_{\text{r1},i},{\tilde{v}}_{\text{r2},j}\right)\\ s.t.\;\tilde{S}\tilde{v}=0\\ {\tilde{v}}_{\text{lb}}\leq \tilde{v}\leq {\tilde{v}}_{\text{ub}}\\ {\tilde{v}}_{r1}={\tilde{v}}_{\text{r1},i}\\ {\tilde{v}}_{r2}={\tilde{v}}_{\text{r2},j}\\ \text{for}\;i=1...m;\;j=1...n \end{array}$$

As a result, the value of the objective function can be plotted over the reactions of interest. In this paper, we used a modified version of the function *phenotypePhasePlane*, which is supplied by the COBRA Toolbox [16]. It takes into account that the reactions of interest are not necessarily uptake reactions, but may also be production reactions of key metabolites or transfer reactions from one time step to another. The modified function is available in the Supplement.

### 2.5. Example Model

**Figure 2:**
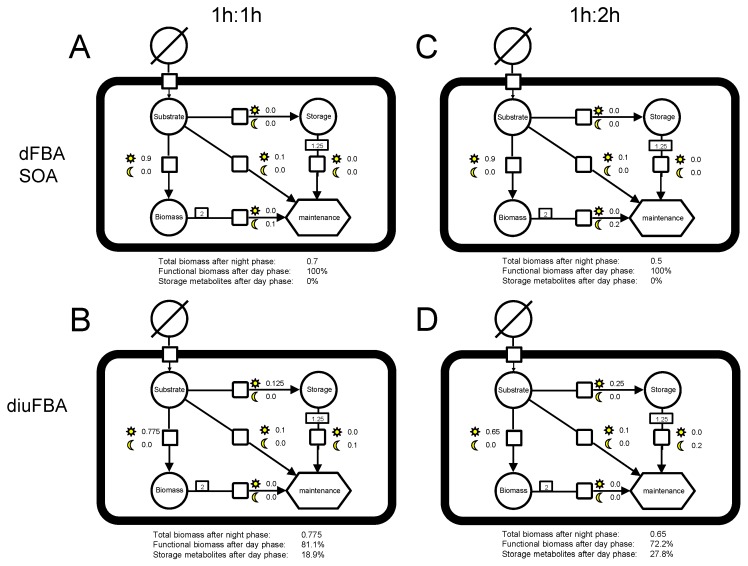
Four instances of the SBGN (Systems Biology Graphical Notation) graph [21] of the example model that demonstrates the principle of the diurnal FBA (diuFBA). The model contains a substrate supply, a storage and a biomass metabolite and a maintenance reaction. Growth and maintenance can be supplied by the substrate, the storage metabolite or biomass, where the storage metabolite is less and the biomass is least efficient. The number next to the Sun indicates the concentration change during substrate availability, while the number next to the Moon indicates concentration change during the starvation phase. The first column shows the optimization results for 1 h of substrate availability and 1 h of starvation. The second column shows the results for 1 h of substrate availability and 2 h of starvation. The first row depicts the results for the dFBA SOA, while the second row depicts the results for the diuFBA.

An abstract example model was created that had the minimal features that are necessary to demonstrate the diuFBA. The model is depicted in Figure 2. The model consists of a substrate supply, which can be considered as photons in a photosynthetic scenario, a storage metabolite and functional biomass, as well as a maintenance reaction. All three components can be used to satisfy maintenance, but with different efficiencies. The use of biomass for maintenance depicts starvation if maintenance cannot be satisfied by either substrate or storage metabolites. For the sake of simplicity, biomass is given in the molar amount of its precursor. In stoichiometric models, these amounts of precursors are multiplied with the molar mass of the precursors to get biomass concentrations in grams of dry cell weight. The model is used to simulate a phase of substrate supply, followed by a phase of substrate limitations. In the photosynthetic context of this paper, the substrate supply phase corresponds to the day phase and the substrate limited phase to the night phase.

## 3. Results and Discussion

### 3.1. Genome-Scale Model

The model iEH410 resulting from the analysis of the annotated genome and gap filling comprises 410 reactions and 363 metabolites. The model created consists of five compartments: cytoplasm, mitochondria, chloroplasts and coccolith vesicles and the Golgi apparatus. Most reactions are located in the cytoplasm, mitochondrion and chloroplast, while the coccolith vesicle and the Golgi apparatus only contain reactions related to calcification. The sources for the reactions are depicted in Table 1. Of the 410 reactions, 221 reactions are directly verified from the genome. Gap filling contributed 72 reactions; 18 reactions describe the build-up of biomass components, and six reactions are derived from other literature sources. There are five chemical equilibrium reactions, which are not enzyme catalyzed and can therefore not be found in the genome. Gap-filling reactions were taken from other genome-scale models, namely the *Escherichia coli* reconstruction iAF1260 [4], the *Arabidopsis thaliana* reconstruction AraGEM [7] and the *Chlamydomonas reinhardtii* reconstruction AlgaGEM [8]. The calcification reaction was modeled according to the calcification hypothesis suggested by MacKinder *et al.* [22], and the rate is fixed to literature values [23].

**Table 1:** Categories of reactions included in the metabolic network. Note that reactions can fall into several categories.

Category	No. of Reactions
Derived from annotated genome	221
Derived from literature	9
Gap filling	72
Transport reactions	86
Chemical equilibrium reactions	5
Build-up of biomass precursors	18
**Overlap with other reconstructions**	
iAF1260 [4]	103
AraGEM [7]	60
AlgaGEM [8]	93

The biomass composition was divided into the precursor metabolite proteins, long chain molecules, lipids and low molecular weight molecules [24]. The protein composition is based on their composition in *Chlamydomonas reinhardtii* [8]. Long chain molecules comprise DNA, RNA and polysaccharides. The DNA composition of *E. huxleyi* was taken from the literature [25], and the stoichiometric coefficients of the DNA synthesis in the AlgaGEM model were adopted accordingly. The polysaccharide composition was taken from the AlgaGEM model. The low molecular weight molecules comprise saccharides and co-factors, and the composition of the biomass precursor molecule was taken from AlgaGEM. The composition of fatty acids was taken from the literature [26]. Since the exact pathways of fatty acid and polysaccharide production are not clearly elucidated, yet, gap-filling reactions, which fulfil stoichiometric requirements, were selected. Since recent studies showed that *E. huxleyi* uses mannitol as a storage metabolite [27,28], we included the mannitol pathway described by Obata *et al.* in the model.

The carbon pool inside a cell is often divided into particulate inorganic carbon (PIC) and particulate organic carbon (POC) [29,30]. We consider biomass to be only organic species. This results in the biomass comprising the POC domain, while coccoliths are considered to be in the PIC domain. Furthermore, coccolith production is dependent on the carbon system of the ocean, so it is not a fixed part of the biomass [31].

The model considers biomass compositions for three metabolic scenarios and two environmental conditions: biomass compositions for a 16-h light phase, for an 8-h dark phase and a combined 24-h resulting biomass. Irradiation is given in Einstein (E), which corresponds to the molar amount of photons. The two experimental conditions comprise high (200 μE m^−2^s^−1^) and low (50 μE m^−2^s^−1^) light irradiation conditions.

**Figure 3:**
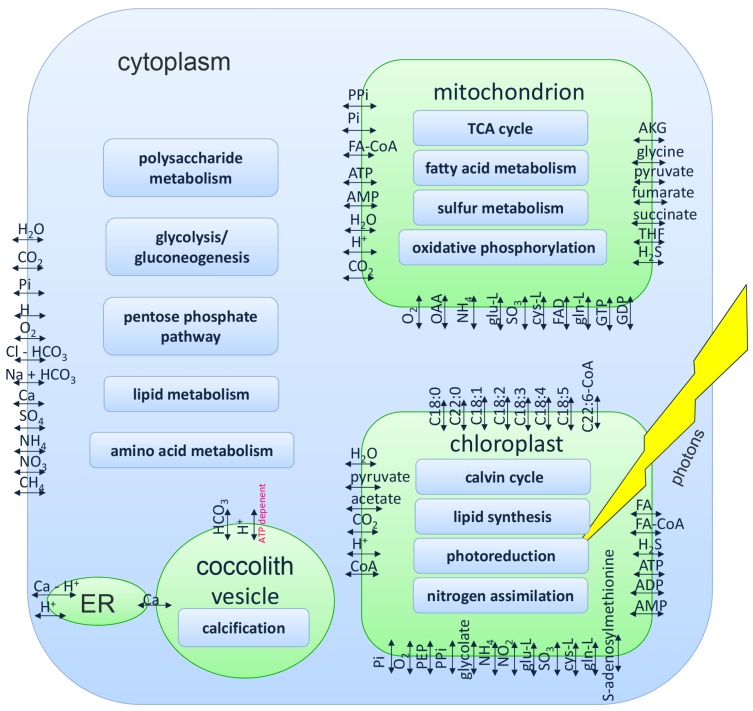
Transport reactions an the localization of major pathways in the iEH410 metabolic reconstruction of *Emiliania huxleyi*. AKG: *α*-ketoglutarate; FA: fatty acid biomass precursor; OAA: oxaloacetic acid; PEP: phosphoenolpyruvate; PPi: pyrophosphate; Pi: phosphate; Cl-HCO_3_: chloride-bicarbonate antiporter; Na+HCO_3_: sodium-bicarbonate symporter; Ca-H^+^: calcium-proton antiporter; C*x*:*y*: fatty acid with *x* carbon atoms and *y* unsaturated bounds; THF: tetrahydrofolate.

The model considers 86 transport reactions between compartments or compartments and the environment. The transport reactions are visualized in Figure 3. Sixty seven of these transport reactions were taken from AlgaGEM; seven describe diffusion across compartment borders; and 12 were described in the annotated genome or literature [22,32,33]. The figure also shows the localization of major metabolic pathways in the compartments of the model.

### 3.2. Analysis of the Example Model

The example model was used to illustrate the flexibility of the diuFBA regarding the biomass composition at the end of the day phase. In a photosynthetic scenario, we can consider the substrate to be photons. Their energy can be used for maintenance processes, or carbon fixation, resulting in the build-up of storage metabolites and biomass.

For the example model, the following two scenarios were chosen: 1 h of 1 M of substrate supply, followed by 1 h without substrate supply. The second scenario comprised 1 h of 1 M of substrate supply, followed by 2 h without substrate supply. The maintenance was set to 0.1 M/h for both scenarios. These scenarios correspond to different day/night length ratios in a photosynthetic scenario.

The results for the dFBA and the diuFBA for the first scenario are shown in the first column of Figure 2. Calculating the optimal flux distribution with two distinct optimization steps similar to the static optimization algorithm of the dFBA [13], all substrates in the first time step are used to create biomass and to satisfy maintenance, resulting in 0.9 M of functional biomass at the end of the first time step (Figure 2A,C). Since no storage metabolites were formed, starvation in the second time step leads to a final biomass concentration of 0.7 M or 0.5 M, respectively.

For the diuFBA in the 1 h:1 h scenario, the optimization resulted in a smaller amount of functional biomass in the first time step (0.775 M), but 0.125 M of storage metabolites were produced. This is just enough to satisfy maintenance demands in the second time step, resulting in a final biomass concentration of 0.775 M. The biomass composition after the first time step is 86.1% functional biomass and 13.9% storage metabolites (Figure 2B).

In the second column, the optimization is repeated for the second scenario with a longer night period. For the diuFBA, this results in an increased production of storage metabolites, altering the biomass composition at the transition from the first to the second phase in favor of more storage metabolite, namely 72.2% functional biomass and 27.8% storage metabolites.

### 3.3. Analysis of the E. huxleyi Model

**Figure 4:**
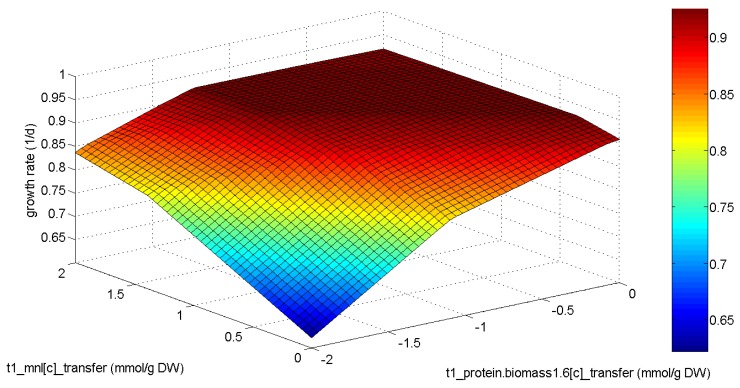
Phenotypic phase plane plot of mannitol production and protein transfer from day to night. Negative numbers mean a transfer from night to day.

The diuFBA was applied to the model of *E. huxleyi*. The biomass composition after a 24-h cycle was considered to be the functional biomass, whereas lipid biomass precursors and mannitol [27,28] were chosen as storage metabolites. The biomass composition at the transition between day and night was calculated by summing up the functional biomass and storage metabolites. A comparison to literature data is shown in Table 2. The method results in a biomass distribution that emphasizes the production of biomass precursors in the daylight time step and the supply of storage metabolites that satisfy the maintenance requirements during the night phase. Mannitol is preferred over lipids as the storage metabolite. The experimentally-observed behavior of increased protein production during the night phase [34] is not depicted. *In silico*, this leads to an increased protein production during the day and a reduced transfer of mannitol, since the carbon for the protein production in the night phase does not need to be transferred. This result can be explained by the phenotypic phase plane (PhPP) plot depicted in Figure 4. In this plot, we consider the effect of a protein production during the night phase and a transfer of this biomass precursor to the day phase, where it is assembled into biomass. We can see that the production during night is possible with less than a 1% reduction of the growth rate (0.924 1/d *vs.* 0.923 1/d). At the same time, the production of mannitol during the day needs to be increased to provide the resources for protein production during the night. This was further illustrated in another diuFBA scenario, in which the protein production during the day was fixed to literature values. The result is also depicted in Table 2. In this scenario, the carbon needed for protein production is transferred to the night phase via mannitol.

**Table 2:** Biomass carbon distribution in pg C /cell after a light phase of 16 h, calculated from Fernandez *et al.* [34], compared to the optimal biomass composition at the same time calculated via diuFBA. For the third column, the protein production was fixed to the literature value.

Biomass Component	Literature	diuFBA	diuFBA (Protein fixed)
Growth rate [1/d]	0.81	0.924	0.893
Proteins	1.52	3.96	1.52
Lipids	8.62	6.69	6.69
Long chain molecules	6.74	2.98	2.98
Low molecular weight molecules	7.77	9.17	12.28
Total carbon	24.65	22.80	25.91

**Figure 5:**
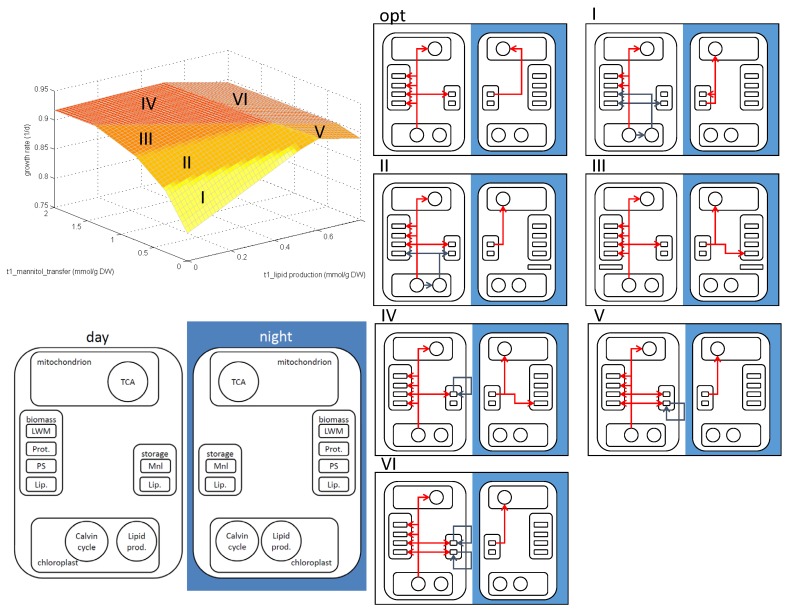
Top left: phenotypic phase plane plot of the stoichiometric model for mannitol transfer from day to night metabolism and lipid production; bottom left: schematic of metabolic pathways and metabolites to categorize the carbon flux distribution in different metabolic modes; right: carbon distribution in the region marked in the phenotypic phase plane (PhPP). Different arrow colors are used for visual distinction only.

This flexibility regarding the choice of storage metabolites was also analyzed using PhPP plots. Figure 5 shows the overall growth rate in a diurnal cycle over the mannitol transfer rate from day to night metabolism and the lipid production rate in the cytoplasm. The plot can be divided into six regions of metabolic activity. Each region corresponds to a metabolic mode, and their carbon distribution patterns are depicted in the images on the right. At the optimal point, all biomass components are produced directly from Calvin cycle metabolites, and mannitol is used as the storage metabolite for the night.

In Mode I, where both mannitol and cytoplasmic lipids are limiting, chloroplastic lipids are produced and exported to the cytoplasm. The chloroplastic lipids are stored for the night, where they are used for energy production and the production of cytoplasmatic lipids. The cytoplasmatic lipids are used during the day for biomass production. In Mode II, enough mannitol is produced during the day to satisfy maintenance during the night. The additionally-transferred mannitol is used to produce cytoplasmatic lipids during the night, which could not be produced during the daytime. Additionally, chloroplastic lipids are still transferred, as the transferred mannitol is not sufficient to satisfy the carbon requirements during the night. Mode III corresponds to a carbon distribution where enough mannitol is available to satisfy the carbon requirements for the night. As not enough cytoplasmatic lipids are produced, yet, mannitol is used to produce them during the night. At the optimal point, the cytoplasmatic lipid production is just high enough to satisfy biomass requirements, and mannitol is used as the sole storage metabolite. Mode IV describes a phase where mannitol production is higher than necessary to fulfil cytoplasmatic lipid production, as well as storage metabolite production for night maintenance. Excessive mannitol is consumed in futile cycles, leading to a decrease in the growth rate. Mode V corresponds to an abundance of cytoplasmatic lipids. These lipids need to be consumed in futile cycles to achieve mass balance. Mode VI corresponds to an overproduction of both cytoplasmatic lipids and mannitol. All excessive metabolites are consumed in futile cycles.

These metabolic modes include the biologically-meaningful Modes II and III, where a combination of lipids and mannitol are used to satisfy both energy storage and functional biomass requirements. Mode I corresponds to an underproduction of both lipids and mannitol that is compensated by an alternative lipid production path. Modes IV, V and VI correspond to an overproduction of possible storage metabolites, which might be used by *E. huxleyi* as a long-term storage, but are consumed due to mass balance constraints.

In the case of the optimal carbon distribution, only mannitol is used as the storage metabolite, and all biomass precursors are produced during day metabolism. We can see in the PhPP plot depicted in Figure 5 that non-optimal (in the sense of maximized biomass production) mannitol-to-lipid ratios are able to result in growth rates found in the literature [30]. As the literature points to a combination of mannitol and lipids as storage metabolites [28], a combination of Modes II and III might describe the actual metabolism.

Although it would have been interesting to compare the results of the diuFBA with the results of the dFBA algorithm, as was done in the example model, this cannot be easily accomplished. In the second phase, the optimization problem for the example model was only solvable due to the starvation term that allowed the conversion of biomass into energy for maintenance. Such a reaction can be easily postulated for a proof-of-principle model, but for real organisms, the response to substrate limitation is much more complex.

## 4. Conclusions

We used the available data of the annotated genome of the *E. huxleyi* strain CCMP 1516, an organism with a strong impact on the global carbon cycle, to create the genome-scale model iEH410. It can be used to determine the energy balance of the organism in periodically-changing light conditions. Understanding the energy distribution for the cellular processes, the build-up of storage metabolites and substrate requirements also makes this organism more accessible for biotechnological purposes. Direct applications of the model include the possibility to map transcriptomic data to the model. Furthermore, the model in combination with the diuFBA approach allows the search for optimized growth media by identifying bottlenecks in the metabolism of *E. huxleyi*.

Regarding the high genetic variability of *E. huxleyi* strains, Read *et al.* point out that no single strain can be considered to be the “typical” *E. huxleyi* strain [3]. This might be relevant considering the many gap filling reactions presented in this paper. While the proteins of some reactions might not be available in CCMP 1516, they might be available in other strains.

A common problem in the analysis of stoichiometric models is the ambiguity of the solution. Methods analyzing this flexibility usually require an optimization problem to be solved repeatedly. In this case, the advantageous formulation of the presented optimization problem as a convex program allows one to derive a quick solution even for large-scale models. Moreover, the resulting optimization problem is in structure identical to the classical flux balance problem, making all methods developed for the classical FBA, such as the phenotypic phase plane analysis, flux variability analysis or elementary modes, easily applicable.

The evolutionary optimality criterion for photosynthetic organisms differs from that of heterotrophic organisms: while the latter have to react to unpredictable substrate availabilities, the former adapted to a deterministic pattern of periodically-changing energy availability. Therefore, a strictly-stationary approach, like the classical FBA algorithm, is unable to correctly depict the evolutionary conditions of photosynthetic organisms. Workarounds, such as different biomass compositions for day and night metabolism, are able to describe measurement data [8], but lack the ability to predict the biomass composition based on the categorization into functional biomass (cell structure, genome, transcriptome and proteome) and storage metabolites. The static optimization approach of the dynamical flux balance analysis [13] can be used to describe time-varying environmental conditions but cannot depict the build-up of storage metabolites as a result of the optimization problem, as the optimization problem is only solved for each time step, not for an overall production goal. The dynamic optimization approach presented in the same paper is able to depict these overall objectives, but results in a non-linear programming problem. Therefore, a repeated solution of the problem, as is necessary, e.g., for the generation of PhPP or a flux variability analysis, can be very time consuming.

The diuFBA approach generalizes the FBA formalism presented by Cheung *et al.* [15]. The reaction vector is considered to consist of concentration changes instead of fluxes. These concentration changes are calculated by integrating the fluxes over the length of the corresponding phase. This time integration allows phases of unequal duration, since the integrated concentration change is no longer dependent on the length of the phase. Furthermore, concentration changes allow the calculation of the biomass composition at the transition between the day and night phase by adding the amount of accumulated storage metabolites to the produced functional biomass components.

The script published in Supplementary S2 can be adapted for the automatic generation and analysis of a diuFBA model in combination with the COBRA Toolbox, while the model by Cheung *et al.* was assembled manually.

Another important extension of the formalism by Cheung *et al.* is the shortened transfer matrix $\tilde{T}$. It allows the consideration of long-term storage by offering a way to export storage metabolites out of the diurnal cycle. Although this feature was not utilized for the analysis of *E. huxleyi*, it might be important for the analysis of higher plants, where metabolites produced in leafs are consumed and stored in other parts of plants.

With the diuFBA, the conflicting goals of functional biomass and storage metabolite production can be described as part of the optimization problem to produce as much biomass as possible while producing just as much storage metabolites as necessary. As shown in the example model, the optimal biomass composition is now the result of the optimization process and no longer a constraint imposed on the basis of measurement data. Moreover, the biomass composition is variable depending on the environmental conditions. As demonstrated with the example model, the ratio of functional and storage metabolites is dependent on the duration of the light and the dark phase. If we applied classical FBA methods, where storage metabolites are included into the biomass function, every new experimental condition would require us to manually adjust the biomass function.

The diuFBA predicts for *E. huxleyi* that proteins are produced during the light phase, but measurements show that the production is distributed over the diurnal cycle, with more production during the dark phase. This discrepancy can be elucidated using PhPP plots of the protein transfer from day to night, representing the ratio of proteins produced in the according phases. This analysis shows that a considerable amount of protein can be produced during the night phase with only minimal influence on the growth rate. This shows the metabolic flexibility regarding protein production. Flexibility regarding the choice of the storage metabolite can be analyzed using the PhPP plot of mannitol and lipids, depicted in Figure 5. There, we can see that the set of storage metabolite production rates that result in less than a 10% reduction of the growth rate is still considerable. Some of the modes are considered unrealistic due to futile cycles burning up storage metabolites to keep the mass balance. If these storage metabolites are used for long-term storage instead of being burned up, even these modes could be considered realistic. At the optimal point, mannitol is favored over lipids as carbon storage due to its energetic efficiency. However, using more lipids as storage (Mode IV, Figure 5) results in only a small loss of growth rate. Recent studies by Tsuji *et al.* suggest that *E. huxleyi* uses this flexibility by producing lipids as storage metabolites instead of relying on mannitol [28]. This illustrates the importance of methods analyzing the solution space around the optimal solution, such as PhPP plots. It allows one to explore non-optimal solutions, which might be preferred due to the effects that cannot be easily depicted by a constraint-based model, such as solubility or stress resistance.

The model and simulation approach can now describe the metabolic processes in wild-type *E. huxleyi* CCMP 1516 under a circadian rhythm. These insights might prove useful for metabolic engineering, where they can be used to divert resources to economically-interesting products, such as highly-structured calcium carbonate in the form of coccoliths or lipids for nutritional/feedstock supplements or petrochemical replacements [3].

## Supplementary Materials

Supplementary materials can be accessed at: http://www.mdpi.com/2218-1989/5/4/659/s1.
