# Supplementary material for: Modeling and Simulation of Optimal Resource Management during the Diurnal Cycle in Emiliania huxleyi by Genome-Scale Reconstruction and an Extended Flux Balance Analysis Approach
Source: Metabolites. 2015 Oct 28;5(4):659–76. doi: 10.3390/metabo5040659 (PMC4693189; doi:10.3390/metabo5040659)
Supplement: Supplementary file 1 [file metabolites-05-00659-s001.zip › S3_iEH410_model_equations.pdf]

Reaction ID	Full reaction name	Equation	diuFBA result (day)	diuFBA result (night)
+T_H2O_c_er	H2O transport (cytoplasma to ER)	h2o[c] <=> h2o[r]	0,079	0,000
4R5AU.SYNT2	5-Amino-6-(1-D-ribitylamino)uracil Synthetase	5 h2o[c] + nadph[c] + gtp[c] -> nadp[c] + for[c] + ppi[c] + nh3_[c] + 4r5au[c] + pi[c] + h[c]	0,001	0,000
AAT	aspartate aminotransferase	asp-L[c] + akg[c] <=> oaa[c] + glu-L[c]	-0,906	0,000
AB.AKG.AMINOTRANS	4-aminobutanoate:2-oxoglutarate aminotransferase	akg[c] + 4abut[c] <=> glu-L[c] + sucsal[c]	0,000	0,000
ACALTRANSF	acetaldehydetransferase (decarboxylating)	h[c] + pyr[c] + 2obut[c] <=> co2[c] + 2ahbut[c]	0,040	0,000
ACO	Aconitase	cit[m] <=> icit[m]	0,446	0,704
ACS-H	acetyl-CoA synthetase (chloroplastide)	ac[h] + atp[h] + coa[h] <=> amp[h] + ppi[h] + accoa[h]	3,891	0,000
ACS-M	mitochondrial Acetyl-Coa Synthetase (Ligase)	coa[m] + ac[m] + atp[m] <=> amp[m] + ppi[m] + accoa[m]	-3,885	0,000
ADCHO.LYASE	aminodeoxychorismate lyase	4adcho[c] -> h[c] + pyr[c] + 4abz[c]	0,001	0,000
ADCHO.SYNT	aminodeoxychorismate synthase	chor[c] + gln-L[c] -> glu-L[c] + 4adcho[c]	0,001	0,000
ADN.AMINOHYD	adenosine aminohydrolase	nh3_[c] + ins[c] <=> h2o[c] + adn[c]	0,188	0,000
ADN.KIN	adenosine kinase	adn[c] + atp[c] -> h[c] + adp[c] + amp[c]	0,188	0,000
AICAR.SYNT	adenylosuccinate lyase (aicar Synthese)	25aics[c] <=> fum[c] + aicar[c]	0,225	0,000
AL.GLX.TRANSAM	alanine glyoxylate transaminase	glx[c] + ala-L[c] -> pyr[c] + gly[c]	0,000	0,000
ALAC.SYNT3	acetolactate synthase	h[c] + 2 pyr[c] -> co2[c] + alac-S[c]	0,175	0,000
ALACREDISO	acetolactate reductoisomerase	alac-S[c] <=> oxbutat[c]	0,175	0,000
ALDH	aldehyde dehydrogenase (NADP+ / NAD+)	h2o[c] + nad[c] + acald[c] <=> 2 h[c] + nadh[c] + ac[c]	-0,675	0,000
ALDH-NADP	aldehyde dehydrogenase (NADP+)	h2o[c] + nadp[c] + acald[c] <=> nadph[c] + 2 h[c] + ac[c]	0,675	0,000
ALK.PHOS	alkaline phosphatase	3 h2o[c] + ahdt[c] -> 3 pi[c] + 2 h[c] + dhnpt[c]	0,001	0,000
ALT	alanine transaminase	glu-L[c] + pyr[c] <=> akg[c] + ala-L[c]	0,339	0,000
AMP.DEAM.ir	AMP deaminase	h2o[c] + amp[c] -> nh3_[c] + imp[c]	0,000	0,000
AMP.KIN	adenylate kinase	atp[c] + amp[c] <=> 2 adp[c]	1,213	0,000
ARAB.ISO	L-arabinose isomerase	rbl-L[c] <=> arab-L[c]	0,745	0,000
ARGINASE.irrev	arginase	h2o[c] + arg-L[c] -> orni[c] + urea[c]	0,000	0,000
ARGSUCLY	argininosuccinate lyase	argsuc[c] <=> fum[c] + arg-L[c]	0,186	0,000
ARGSUCSYNT	argininosuccinate synthase	asp-L[c] + atp[c] + citr-L[c] <=> ppi[c] + h[c] + amp[c] + argsuc[c]	0,186	0,000
AS	asparagine synthetase (glutamine-hydrolysing)	nh3_[c] + asp-L[c] + atp[c] <=> ppi[c] + amp[c] + asn-L[c]	0,084	0,000
ASP.CBP.TRANS	aspartate carbamoyltransferase	asp-L[c] + cbp[c] -> pi[c] + h[c] + cbasp[c]	0,057	0,000
ASP.KIN	aspartate kinase	asp-L[c] + atp[c] -> adp[c] + 4pasp[c]	0,270	0,000
ASPSA.DH	aspartate-semialdehyde dehydrogenase	nadph[c] + h[c] + 4pasp[c] <=> nadp[c] + pi[c] + aspsa[c]	0,270	0,000
BETA-OX3-M	beta oxidation of fatty acid -coa.replacement (Mitochondria)	8.123 coa[m] + 8.123 nad[m] + fa-coa.replace_i[m] + 8.123 h2o[m] + 5.28 fad[m] -> 9.123 accoa[m] + 8.123 nadh[m] + 8.123 h[m] + 5.28 fadh2[m]	0,000	0,000
BETA.GAL(300)	beta-galactosidase; Galactan galactohydrolase	299 h2o[c] + udp.galactan(300)[c] -> 299 gal[c] + udpgal[c]	0,000	0,000
BIOM_LOG_200µE_16h_light_neutralcharge_final	biomass reaction for E.huxleyi in exp growth phase with high Irradiation during light phase (200µE /m^2*s)	1.006 h[c] + main.biomass1[c] + 0.44222 protein.biomass1.6[c] + 0.58827 lipid.biomass5.[c] + 0.71108 lcm.biomass2.1[c] + 1.372 lmwm.biomass[c] -> biomass_log_200_16h_light_neutrcharge_final2[c]	0,000	0,000
BIOM_LOG_200µE_24h_neutralcharge_final	biomass reaction for E.huxleyi in exp growth phase with high Irradiation during light phase (200µE /m^2*s)	0.98997 h[c] + main.biomass1[c] + 1.3386 protein.biomass1.6[c] + 0.55126 lipid.biomass5.[c] + 0.36632 lcm.biomass2.1[c] + 1.5383 lmwm.biomass[c] -> biomass_log_200_24h_neutrcharge_final[c]	0,924	0,000
BIOM_LOG_50µE_16h_light_neutralcharge_final	biomass reaction for E.huxleyi in exp growth phase with low Irradiation during 16h light phase (50µE /m^2*s)	0.98223 h[c] + main.biomass1[c] + 0.79948 protein.biomass1.6[c] + 0.63279 lipid.biomass5.[c] + 0.49194 lcm.biomass2.1[c] + 1.4736 lmwm.biomass[c] -> biomass_log_50_16h_neutrcharge_final[c]	0,000	0,000
BIOM_LOG_50µE_24h_neutralcharge_final	biomass reaction for E.huxleyi in exp growth phase with high Irradiation during light phase (200µE /m^2*s)	0.72836 h[c] + main.biomass1[c] + 1.8695 protein.biomass1.6[c] + 0.67052 lipid.biomass5.[c] + 0.30087 lcm.biomass2.1[c] + 1.2306 lmwm.biomass[c] -> biomass_log_50_24h_neutrcharge_final[c]	0,000	0,000

C14:0_FS.SYNT2-H	C14:0 fatty acid synthesis (chloroplastide)	6 atp[h] + 7 accoa[h] + 12 nadph[h] + h2o[h] + 5 h[h] -> 7 coa[h] + 12 nadp[h] + 6 adp[h] + 6 pi[h] + 14:0_FS[h]	0,067	0,000
C16:0_FS.SYNT-H	C16:0 fatty acid synthesis (chloroplastide)	7 atp[h] + 8 accoa[h] + 14 nadph[h] + h2o[h] + 6 h[h] -> 8 coa[h] + 14 nadp[h] + 7 adp[h] + 7 pi[h] + hdca[h]	0,034	0,000
C18:0_FS.SYNT-H	C18:0 fatty acid synthesis (chloroplastide)	8 atp[h] + 9 accoa[h] + 16 nadph[h] + h2o[h] + 7 h[h] -> 9 coa[h] + 16 nadp[h] + 8 adp[h] + 8 pi[h] + ocdca[h]	0,215	0,000
C18:1.DESAT-H	(18:1) desaturase (chloroplastide)	h[h] + ocdca[h] + o2[h] + nadh[h] <=> 2 h2o[h] + nad[h] + ocdcea[h]	0,212	0,000
C18:2.DESAT-H	(18:2) desaturase (chloroplastide)	h[h] + o2[h] + nadh[h] + ocdcea[h] <=> 2 h2o[h] + nad[h] + ocdciea[h]	0,122	0,000
C18:3.DESAT2-H	(18:3) desaturase (chloroplastide)	h[h] + o2[h] + nadh[h] + ocdciea[h] <=> 2 h2o[h] + nad[h] + ocdctria[h]	0,099	0,000
C18:4.DESAT-H	(18:4) desaturase (chloroplastide)	h[h] + o2[h] + nadh[h] + ocdctria[h] <=> 2 h2o[h] + nad[h] + ocdctetra[h]	0,074	0,000
C18:5.DESAT-H	(18:5) desaturase (chloroplastide)	h[h] + o2[h] + nadh[h] + ocdctetra[h] <=> 2 h2o[h] + nad[h] + ocdcpenta[h]	0,042	0,000
C22:0_FS.SYNT-H	(22:0) fatty acid synthesis (chloroplastide)	10 atp[h] + 11 accoa[h] + 20 nadph[h] + h2o[h] + 9 h[h] -> 11 coa[h] + 20 nadp[h] + 10 adp[h] + 10 pi[h] + c22:0[h]	0,110	0,000
C22:6.DESAT-H	(22:6) desaturase (chloroplastide)	6 h[h] + 6 o2[h] + 6 nadh[h] + c22:0[h] <=> 12 h2o[h] + 6 nad[h] + c22:6[h]	0,110	0,000
CALCI-2-O	calcification	hco3[o] + ca2[o] <=> h[o] + caco3[o]	23,520	11,760
CBPSYNT	carbamoylphosphat synthetase	h2o[c] + nh3_[c] + co2[c] + 2 atp[c] <=> pi[c] + 2 h[c] + 2 adp[c] + cbp[c]	0,243	0,000
CHOR.SYNT4	chorismate synthesis	nadph[c] + atp[c] + e4p[c] + 2 pep[c] <=> nadp[c] + 4 pi[c] + chor[c] + adp[c]	0,047	0,000
CHORM	chorismate mutase	chor[c] -> pphn[c]	0,043	0,000
CO2c-m	CO2 transport via diffusion (cytoplasma to mitochondrium)	co2[c] <=> co2[m]	-4,790	-2,113
CO2c-p	CO2 transport via diffusion (cytoplasma to chloroplast)	co2[c] <=> co2[h]	41,011	0,000
CO2trex	CO2 transport via diffusion (extracellular to cytoplasma)	co2[e] <=> co2[c]	61,087	9,647
CS-M	citrate synthase	accoa[m] + h2o[m] + oaa[m] -> cit[m] + coa[m] + h[m]	0,446	0,704
CTPS1	CTP synthase	nh3_[c] + atp[c] + utp[c] -> pi[c] + h[c] + adp[c] + ctp[c]	0,035	0,000
CTPS3	CTP synthase (glutamine)	h2o[c] + gln-L[c] + atp[c] + utp[c] -> pi[c] + 2 h[c] + glu-L[c] + adp[c] + ctp[c]	0,000	0,000
CYSDS	cysteine Desulfhydrase	h2o[c] + cys-L[c] -> pyr[c] + h2s[c] + nh4[c]	0,000	0,000
CYSSYNT-M	cysteine synthetase (mitochondrion)	acser[m] + h2s[m] <=> ac[m] + h[m] + cys-L[m]	0,006	0,000
CYSTASYNT	cystathionine beta-synthase	ser-L[c] + hcys-L[c] <=> h2o[c] + cyst-L[c]	-0,003	0,000
CYSTHIO	cystathionase	h2o[c] + cyst-L[c] <=> 2obut[c] + cys-L[c] + nh4[c]	-0,003	0,000
CYTK1	cytidylate kinase (CMP)	atp[c] + cmp[c] <=> adp[c] + cdp[c]	0,085	0,000
CYTK2	cytidylate kinase (dCMP)	atp[c] + dcmp[c] <=> adp[c] + dcdp[c]	0,001	0,000
DAPDC	diaminopimelate decarboxylase	h[c] + 26dap-M[c] -> co2[c] + lys-L[c]	0,022	0,000
DAPE	diaminopimelate epimerase	26dap-LL[c] <=> 26dap-M[c]	0,022	0,000
DB4PS	3.4-Dihydroxy-2-butanone-4-phosphate synthase	ru5p-D[c] -> for[c] + h[c] + db4p[c]	0,001	0,000
DCTP.DEAM	dCMP Deaminase	h2o[c] + dcmp[c] <=> nh3_[c] + dump[c]	-0,001	0,000
DHAD	dihydroxy-acid dehydratase	23dhmb[c] <=> h2o[c] + 3mob[c]	0,175	0,000
DHAHL	dihydroxy-acid hydrolyase	23dhmp[c] <=> h2o[c] + 3mop[c]	0,040	0,000
DHFR	dihydrofolate reductase	nadph[c] + h[c] + dhf[c] <=> nadp[c] + thf[c]	0,002	0,000
DHFS	dihydrofolate synthase	glu-L[c] + atp[c] + dhpt[c] -> pi[c] + h[c] + adp[c] + dhf[c]	0,001	0,000
DHOOAT.DH	dihydroorotate dehydrogenase	nad[c] + dhor-S[c] <=> h[c] + nadh[c] + orot[c]	0,057	0,000
DHORTS	dihydroorotase	h2o[c] + dhor-S[c] <=> h[c] + cbasp[c]	-0,057	0,000
DHPS	dihydropteroate synthase	4abz[c] + dhpterin.pp[c] -> ppi[c] + dhpt[c]	0,001	0,000
DIAM.PIMEL.SYNT	LL-2.6-Diaminopimelate synthase	h[c] + glu-L[c] + pyr[c] + nadh[c] + aspsa[c] <=> h2o[c] + akg[c] + nad[c] + 26dap-LL[c]	0,022	0,000
DIHY.ALDO	dihydroneopterin aldolase	dhnpt[c] -> gcald[c] + dhpterin[c]	0,001	0,000
DMLZ.SYNT	"	4r5au[c] + db4p[c] -> 2 h2o[c] + pi[c] + dmlz[c]	0,001	0,000

DTMPK	dTMP kinase	atp[c] + dtmp[c] <=> adp[c] + dtdp[c]	0,001	0,000
DUTPDP	dUTP diphosphatase	h2o[c] + dutp[c] -> ppi[c] + h[c] + dump[c]	0,000	0,000
ENO	enolase	2pg[c] <=> h2o[c] + pep[c]	4,098	-0,352
ENO1-H	enolase (chloroplastide)	2pg[h] <=> h2o[h] + pep[h]	2,288	1,056
ETHA.OXI	ethanolamine oxidase	h2o[c] + o2[c] + etha[c] -> nh3_[c] + h[c] + gcald[c] + h2o2[c]	0,000	0,000
EXP_COCC	Coccolith Export	caco3[o] ->	23,520	11,760
EX_LCM.2.1	Allocation of biomass precorsor lcm.biomass2.1	lcm.biomass2.1[e] <=>	0,000	0,000
EX_LIPID5	Allocation of biomass precorsor lipid.biomass4	lipid.biomass5.[e] <=>	0,000	0,000
EX_LMWM	Allocation of biomass precorsor lmwm.biomass	lmwm.biomass[e] <=>	0,000	0,000
EX_ca2(e)	Calcium exchange	ca2[e] <=>	-23,520	-11,760
EX_co2(e)	CO2 exchange	co2[e] <=>	-61,087	-9,647
EX_h(e)	H+ exchange	h[e] <=>	49,160	23,520
EX_h2o(e)	H2O exchange	h2o[e] <=>	-50,695	-9,295
EX_hn(e)	Photon exchange	hn[e] <=>	-387,840	0,000
EX_na1(e)	Sodium exchange	na1[e] <=>	0,000	0,000
EX_nh4(e)	Ammonia exchange	nh4[e] <=>	-3,615	0,000
EX_no3(e)	Nitrate exchange	no3[e] <=>	0,000	0,000
EX_o2(e)	O2 exchange	o2[e] <=>	40,019	-2,289
EX_pi(e)	Phosphate exchange	pi[e] <=>	-0,669	0,000
EX_so4(e)	Sulfate exchange	so4[e] <=>	-0,023	0,000
Ex_HCO3(e)	HCO3 exchange	hco3[e] <=>	0,000	0,000
F6PP	D-fructose 6-phosphate phosphatase	h2o[c] + f6p[c] -> pi[c] + fru[c]	0,000	0,000
FA.REPL.COA.SYNT_d	Fatty Acid Replacement-CoA Synthetase	atp[h] + coa[h] + fa.replace_d[h] -> amp[h] + ppi[h] + fa-coa.replace_i[h]	0,425	0,000
FA.REPL_16_3_f	Fatty Acid Replacement, CO2 concentration [16,3 mmol/1000L]	0.158 14:0_FS[h] + 0.079 hdca[h] + 0.006 ocdca[h] + 0.211 ocdcea[h] + 0.056 ocdciea[h] + 0.057 ocdctria[h] + 0.075 ocdctetra[h] + 0.099 ocdcpenta[h] + 0.259 c22:6[h] -> fa.replace_d[h]	0,426	0,000
FBA	fructose-bisphosphate aldolase	fdp[c] <=> dhap[c] + g3p[c]	-3,320	0,352
FBA-H	fructose-bisphosphate aldolase (Chloroplast)	fdp[h] <=> g3p[h] + dhap[h]	0,000	0,000
FBA2	fructose-bisphosphate aldolase	s17bp[c] <=> e4p[c] + dhap[c]	0,000	0,000
FBA2-H	fructose-bisphosphate aldolase (Chlorplast)	s17bp[h] <=> dhap[h] + e4p[h]	-27,402	0,000
FBP	fructose-bisphosphatase	h2o[c] + fdp[c] -> pi[c] + f6p[c]	3,320	0,000
FBP-H	fructose-bisphosphatase	h2o[h] + fdp[h] -> pi[h] + f6p[h]	0,000	0,000
FER.OXRED-H	Ferredoxin:NADP+ oxidoreductase (Chloroplastides)	h[h] + nadp[h] + 2 redfer1[h] <=> nadph[h] + 2 oxfer[h]	-8,993	-1,056
FER.OXRED-M	Ferredoxin:NADP+ oxidoreductase (Mitochondrion)	h[m] + 2 redfer1[m] + nadp[m] <=> nadph[m] + 2 oxfer[m]	8,975	1,056
FMN.HYD	FMN Phosphatase; riboflavin-5-phosphate phosphohydrolase	h2o[c] + fmn[c] -> pi[c] + ribflv[c]	0,000	0,000
FMNAT	FMN adenylyltransferase	h[c] + atp[c] + fmn[c] -> ppi[c] + fad[c]	0,000	0,000
FOR.DH	Formate dehydrogenase (oxidoreductase)	for[c] + nad[c] <=> co2[c] + nadh[c]	-0,449	0,000
FTHF.SYNT	10-formyltetrahydrofolate synthetase	for[c] + atp[c] + thf[c] -> pi[c] + adp[c] + 10fthf[c]	0,452	0,000
FUM-M	fumarase	h2o[m] + fum[m] <=> mal-L[m]	0,411	0,704
G3P.DH-H	glyceraldehyde-3-phosphate dehydrogenase (Chloroplast)	nadp[h] + pi[h] + g3p[h] <=> nadph[h] + h[h] + 13dpg[h]	-81,038	1,056
G3PD2	glycerol-3-phosphate dehydrogenase (NADP)	nadp[c] + glyc3p[c] <=> nadph[c] + h[c] + dhap[c]	0,000	0,000
G3PDH-H	glyceraldehyde-3-phosphate dehydrogenase (Chloroplast)	pi[h] + nad[h] + g3p[h] <=> h[h] + nadh[h] + 13dpg[h]	1,213	0,000

G3PDHirr	glyceraldehyde-3-phosphate dehydrogenase (NADP+)	h2o[c] + nadp[c] + g3p[c] -> nadph[c] + 2 h[c] + 3pg[c]	0,000	0,000
G6PDH2-H	glucose 6-phosphate dehydrogenase (Chloroplast)	nadp[h] + g6p[h] <=> nadph[h] + h[h] + 6pgl[h]	0,000	0,000
G6PDH2r	glucose 6-phosphate dehydrogenase	nadp[c] + g6p[c] <=> nadph[c] + h[c] + 6pgl[c]	0,000	0,000
GALKr	galactokinase	atp[c] + gal[c] <=> h[c] + adp[c] + gal1p[c]	-0,991	0,000
GALUrev	UTP-glucose-1-phosphate uridylyltransferase	h[c] + utp[c] + g1p[c] <=> ppi[c] + udpg[c]	1,795	0,000
GAPD	glyceraldehyde-3-phosphate dehydrogenase	pi[c] + nad[c] + g3p[c] <=> h[c] + nadh[c] + 13dpg[c]	4,098	-0,352
GARFT.ir	phosphoribosylglycinamide formyltransferase (irreversible)	10fthf[c] + gar[c] -> h[c] + thf[c] + fgam[c]	0,225	0,000
GAl1P.UDP.TRANS	galactose-1-phosphate uridylyltransferase (cytoplasma)	h[c] + utp[c] + gal1p[c] <=> ppi[c] + udpgal[c]	-0,991	0,000
GCAL.DH	Glycolaldehyde dehydrogenase	h2o[c] + nad[c] + gcald[c] -> 2 h[c] + nadh[c] + glyclt[c]	0,001	0,000
GHMT2r	glycine hydroxymethyltransferase. 1	ser-L[c] + thf[c] <=> h2o[c] + gly[c] + mlthf[c]	0,170	0,000
GHMT2r-M	glycine hydroxymethyltransferase (mitochondrion)	thf[m] + ser-L[m] <=> h2o[m] + mlthf[m] + gly[m]	-0,006	0,000
GK1	guanylate kinase (GMP:ATP)	atp[c] + gmp[c] <=> adp[c] + gdp[c]	0,039	0,000
GLNS	glutamine synthetase	glu-L[c] + atp[c] + nh4[c] -> pi[c] + h[c] + gln-L[c] + adp[c]	0,000	0,000
GLNS-H	glutamine synthetase (chloroplast)	atp[h] + glu-L[h] + nh4[h] -> h[h] + adp[h] + pi[h] + gln-L[h]	0,000	0,000
GLNS-M	glutamine synthetase (mitochondrion)	atp[m] + glu-L[m] + nh4[m] -> h[m] + adp[m] + gln-L[m] + pi[m]	0,000	0,000
GLUC(300).HYD	1.3-beta-D-glucan glucanohydrolase	300 h2o[c] + 13glucan(300)[c] -> 300 glc-D[c]	0,000	0,000
GLUC.SYN(300)	1.3-beta-glucan synthase (300)	299 udpg[c] + glc-D[c] -> h2o[c] + 299 h[c] + 13glucan(300)[c] + 299 udp[c]	0,002	0,000
GLUCYS	gamma-glutamylcysteine synthetase	glu-L[c] + atp[c] + cys-L[c] -> pi[c] + h[c] + adp[c] + glucys[c]	0,000	0,000
GLUDC	Glutamate Decarboxylase	h[c] + glu-L[c] -> 4abut[c] + co2[c]	0,000	0,000
GLUN.rev	glutaminase (reversible)	h2o[c] + gln-L[c] <=> glu-L[c] + nh4[c]	-10000,000	-10000,000
GLUPRT	glutamine phosphoribosyldiphosphate amidotransferase	h2o[c] + gln-L[c] + prpp[c] -> ppi[c] + glu-L[c] + pram[c]	10000,000	10000,000
GLUPRT.rev	glutamine phosphoribosyldiphosphate amidotransferase	h2o[c] + gln-L[c] + prpp[c] <=> ppi[c] + glu-L[c] + pram[c]	-9999,775	-10000,000
GLURING	Glutamate ring building	glu5sa[c] <=> h2o[c] + h[c] + 1pyr5c[c]	0,058	0,000
GLUSYN.irrev	glutamate synthase (NADPH)	nadph[c] + h[c] + akg[c] + gln-L[c] -> nadp[c] + 2 glu-L[c]	9999,407	10000,000
GLUT.P.RED	beta-glutamylphosphate reductase	nadph[c] + h[c] + glu5p[c] <=> nadp[c] + pi[c] + glu5sa[c]	0,244	0,000
GLUTKIN	glutamate 5-kinase	glu-L[c] + atp[c] <=> adp[c] + glu5p[c]	0,244	0,000
GLUTSYNT	Glutamate dehydrogenase (NadpH.) ; Glutamatesynthesis	nadph[c] + h[c] + akg[c] + nh4[c] <=> h2o[c] + nadp[c] + glu-L[c]	-10000,000	-10000,000
GLUTSYNT2	Glutamate dehydrogenase (NadH.) ; Glutamatesynthesis	h[c] + akg[c] + nadh[c] + nh4[c] <=> h2o[c] + glu-L[c] + nad[c]	2,846	0,000
GLY.CLVG-M	glycine cleavage (Mitochondrion)	nad[m] + thf[m] + gly[m] -> nadh[m] + co2[m] + mlthf[m] + nh4[m]	0,006	0,000
GLYALD.OXIRED	Glyceraldehyde oxidoreductase (NAD+)	h2o[c] + nad[c] + glyald[c] <=> 2 h[c] + nadh[c] + glyc-R[c]	0,000	0,000
GLYAT	glycine C-acetyltransferase	gly[c] + accoa[c] <=> coa[c] + 2aobut[c]	-0,102	0,000
GLYC-R.KIN	glycerate 3-kinase	atp[c] + glyc-R[c] <=> h[c] + adp[c] + 3pg[c]	0,000	0,000
GLYC.OXIRED	Glycerol oxidoreductas	nad[c] + glyc[c] <=> h[c] + nadh[c] + glyald[c]	0,000	0,000
GLYCL	Glycine Cleavage System	gly[c] + nad[c] + thf[c] -> co2[c] + nadh[c] + nh4[c] + mlthf[c]	0,000	0,000
GLYCLT.DH	glycolate dehydrogenase (glycolate oxidase)	o2[c] + glyclt[c] -> glx[c] + h2o2[c]	0,093	0,000
GMP.REDirr	GMP reductase	nadph[c] + h[c] + gmp[c] -> nadp[c] + nh3_[c] + imp[c]	0,000	0,000
GMP.SYNT	GMP synthase	h2o[c] + gln-L[c] + atp[c] + xmp[c] -> ppi[c] + 2 h[c] + glu-L[c] + amp[c] + gmp[c]	0,039	0,000
GOA	Glycine:2-oxoglutarate aminotransferase	glu-L[c] + glx[c] -> akg[c] + gly[c]	0,093	0,000

GROWTH_log_200_16h_neutralcharge	Exportfunction of biomass (logarithmic growth. high irradiation 200müE in light phase) metabolite	biomass_log_200_16h_light_neutrcharge_final2[c] ->	0,000	0,000
GROWTH_log_200_24h_neutralcharge	Exportfunction of biomass (logarithmic growth. high irradiation 200µE) metabolite	biomass_log_200_24h_neutrcharge_final[c] ->	0,000	0,000
GROWTH_log_50_16h_neutralcharge	Exportfunction of biomass (logarithmic growth. low irradiation 50µE) metabolite	biomass_log_50_16h_neutrcharge_final[c] ->	0,000	0,000
GROWTH_log_50_24h_neutralcharge	Exportfunction of biomass (logarithmic growth. low irradiation 50µE) metabolite	biomass_log_50_24h_neutrcharge_final[c] ->	0,000	0,000
GTHOrev	glutathione oxidoreductase	nadph[c] + h[c] + gthox[c] <=> nadp[c] + 2 gthrd[c]	-9999,931	-10000,000
GTHOrev2	glutathione oxidoreductase (nadh)	h[c] + nadh[c] + gthox[c] <=> nad[c] + 2 gthrd[c]	10000,000	10000,000
GTHRD.PEROX	glutathione peroxidase	h2o2[c] + 2 gthrd[c] -> 2 h2o[c] + gthox[c]	0,069	0,000
GTHS	glutathione synthetase	atp[c] + gly[c] + glucys[c] -> pi[c] + h[c] + adp[c] + gthrd[c]	0,000	0,000
GTPCI	GTP cyclohydrolase I	h2o[c] + gtp[c] -> for[c] + h[c] + ahdt[c]	0,001	0,000
H2O.EQ	h2o equilibrium	h[c] + oh[c] <=> h2o[c]	0,001	0,000
HCO3E	HCO3 equilibration reaction	h2o[c] + co2[c] <=> h[c] + hco3[c]	24,461	11,760
HCO3E-M	HCO3 equilibration reaction	h2o[m] + co2[m] <=> h[m] + hco3[m]	0,000	0,000
HEX1	hexokinase (D-glucose:ATP)	atp[c] + glc-D[c] -> h[c] + adp[c] + g6p[c]	0,000	0,000
HEX4	hexokinase (D-mannose:ATP)	atp[c] + man[c] -> h[c] + adp[c] + man6p[c]	0,000	0,352
HISTDH	histidinol dehydrogenase	h2o[c] + 2 nad[c] + histd[c] -> 3 h[c] + 2 nadh[c] + his-L[c]	0,001	0,000
HISTP	histidinol-phosphatase	h2o[c] + hisp[c] -> pi[c] + histd[c]	0,001	0,000
HPPK	7.8-Dihydro-6-hydroxymethylpterin-pyrophosphokinase	atp[c] + dhpterin[c] -> h[c] + amp[c] + dhpterin.pp[c]	0,001	0,000
HSDy	homoserine dehydrogenase (NADPH)	nadp[c] + hom-L[c] <=> nadph[c] + h[c] + aspsa[c]	-0,247	0,000
HSK	homoserine kinase	atp[c] + hom-L[c] -> h[c] + adp[c] + phom[c]	0,247	0,000
HSTPT	histidinol-phosphate transaminase	glu-L[c] + imacp[c] -> akg[c] + hisp[c]	0,001	0,000
IC.DH-M	isocitrate dehydrogenase (NAD) (Mitochondrion)	icit[m] + nad[m] <=> nadh[m] + co2[m] + akg[m]	9,421	1,760
IC.DH1-M	isocitrate dehydrogenase (NADP) (mitochondrion)	icit[m] + nadp[m] <=> co2[m] + nadph[m] + akg[m]	-8,975	-1,056
IGP.SYNT	Imidazole glycerol phosphate synthase	gln-L[c] + prlp[c] <=> h[c] + glu-L[c] + aicar[c] + eig3p[c]	0,001	0,000
IGPDH	imidazoleglycerol-phosphate dehydratase	eig3p[c] -> h2o[c] + imacp[c]	0,001	0,000
ILETA	isoleucine transaminase	akg[c] + ile-L[c] <=> glu-L[c] + 3mop[c]	-0,040	0,000
IMP.SYNT	AICAR formyltransferase (IMP Biosynthesis)	aicar[c] + 10fthf[c] <=> h2o[c] + imp[c] + thf[c]	0,227	0,000
IMPD	IMP dehydrogenase	h2o[c] + nad[c] + imp[c] -> h[c] + nadh[c] + xmp[c]	0,039	0,000
INOST.SYNT	Inositol Synthetase	h2o[c] + g6p[c] -> pi[c] + inost[c]	0,003	0,000
IPMLATDH2	3-isopropylmalate dehydrogenase	nad[c] + 3c2hmp[c] <=> co2[c] + nadh[c] + 4mop[c]	0,102	0,000
IPMLATHYDLY	3-isopropylmalate hydrolyase	3c3hmp[c] <=> 3c2hmp[c]	0,102	0,000
IPMLATSYNT	2-isopropylmalate synthetase	h2o[c] + 3mob[c] + accoa[c] <=> h[c] + coa[c] + 3c3hmp[c]	0,102	0,000
Import_log_200i_8h_dark	Night Metabolism during 8h dark phase of high irradiated (during day) E.hux in log growth [g Metab / (g DCW* hr)]	0.015607 lcm.biomass2.1[e] + 0.0045351 lipid.biomass5.[e] + 0.0020506 lmwm.biomass[e] -> 0.0045351 lipid.biomass5.[c] + 0.015607 lcm.biomass2.1[c] + 0.0020506 lmwm.biomass[c]	0,000	0,000
Import_log_50i_8h_dark	Night Metabolism during 8h dark phase of low irradiated (during day) E.hux in log growth [g Metab / (g DCW* hr)]	0.0045958 lcm.biomass2.1[e] + 0.00012431 lipid.biomass5.[e] + 0.0070444 lmwm.biomass[e] -> 0.00012431 lipid.biomass5.[c] + 0.0045958 lcm.biomass2.1[c] + 0.0070444 lmwm.biomass[c]	0,000	0,000
KARA2	ketol-acid reductoisomerase (2-Acetolactate)	nadph[c] + h[c] + 2ahbut[c] <=> nadp[c] + 23dhmp[c]	0,040	0,000

LCM.BIOM1.6	Long Chain Molecules Biomass	0.33771 h2o[c] + 0.10421 gtp[c] + 0.058616 atp[c] + 0.058616 utp[c] + 0.10421 ctp[c] + 0.0064079 13glucan(300)[c] + 0.0021191 datp[c] + 0.0021191 dttp[c] + 0.0039356 dctp[c] + 0.0039356 dgtp[c] -> 0.33771 ppi[c] + lcm.biomass2.1[c]	0,339	0,000
LCM.DEGRAD	Long Chain Molecules Degradation	lcm.biomass2.1[c] -> 0.0064079 13glucan(300)[c]	0,000	0,000
LEUSYNT	branched-chain aminotransferase (Leucin biosynthesis)	glu-L[c] + 4mop[c] <=> akg[c] + leu-L[c]	0,102	0,000
LIPASE.LIPID.BIOM_final	Lipase of Lipid.biom2.1; lipid composition after 7 days of growth (log)	1.1539 h2o[c] + lipid.biomass5.[c] -> 0.26699 gal[c] + 0.029625 etha[c] + 0.058754 glyc3p[c] + 0.37925 glyc[c] + 0.83705 fa.replace_d[c]	0,000	0,000
LIPID.BIOM3	Lipid biomass (log)	lipid.chloroplast2[c] + -> lipid.biomass5.[c]	0,510	0,000
LIPID.CHLORO-H	Lipid biomass (log) (Chloroplastide)	0.25358 h2o[h] + 0.0036923 fa.replace_d[h] + 0.52305 fa-coa.replace_i[h] + 0.25888 g3p[h] + 0.011987 ctp[h] + 0.033764 udp-sqv[h] + 0.26699 udpgal[h] -> 0.52305 coa[h] + 0.26699 pi[h] + 0.011987 cmp[h] + 0.30075 udp[h] + lipid.chloroplast2[h]	0,510	0,000
LIPID.ER	Lipid biomass (log) (endoplasmatic reticulum)	0.15515 h2o[r] + 0.15515 g3p[r] + 0.3103 fa-coa.replace_i[r] + 0.15515 ctp[r] + 0.13801 ser-L[r] + 0.0051541 inost[r] + 0.32515 amet[r] -> 0.14316 ppi[r] + 0.023975 pi[r] + 0.3103 coa[r] + 0.13801 co2[r] + 0.15515 cmp[r] + 0.32515 hcys-L[r] + lipid.er4[r]	0,510	0,000
LMWM.BIOM	Low Molecular Weight Metabolites Biomass	0.000223 chor[c] + 0.52414 arab-L[c] + 0.6969 gal[c] + 0.000223 thf[c] + 0.000223 ribflv[c] + 0.000223 fad[c] + 0.000223 10fthf[c] + 0.000223 mlthf[c] + 0.32836 man6p[c] + 0.000223 5mthf[c] -> lmwm.biomass[c]	1,422	0,000
LMWM.DEGRAD	Low Molecular Weight Metabolites Degradation	lmwm.biomass[c] -> 0.000223 chor[c] + 0.52414 arab-L[c] + 0.6969 gal[c] + 0.32836 man6p[c]	0,000	0,000
M1PD	mannitol-1-phosphate 5-dehydrogenase	nad[c] + mnl1p[c] <=> h[c] + nadh[c] + f6p[c]	-0,352	0,000
M1Pase_irrev	Mannitol-1-phosphatase	h2o[c] + mnl1p[c] -> pi[c] + mnl[c]	0,352	0,000
MAIN.BIOM2	ATP maintenance (growth-associated)	39.24 h2o[c] + 39.24 atp[c] -> 39.24 pi[c] + 39.24 h[c] + 39.24 adp[c] + main.biomass1[c]	0,924	0,000
MAL.DH	Malate dehydrogenase	nad[m] + mal-L[m] -> nadh[m] + co2[m] + pyr[m]	0,000	0,000
MAN6PI	mannose-6-phosphate isomerase	man6p[c] <=> f6p[c]	-0,467	0,352
MDH-M	malate dehydrogenase	nad[m] + mal-L[m] <=> nadh[m] + h[m] + oaa[m]	0,411	0,704
METAT	methionine adenosyltransferase	h2o[c] + atp[c] + met-L[c] -> ppi[c] + pi[c] + amet[c]	0,166	0,000
METS	methionine synthase	hcys-L[c] + 5mthf[c] -> h[c] + thf[c] + met-L[c]	0,169	0,000
MTHF.RED2ir	methylenetetrahydrofolate reductase (NADPH	nadph[c] + 2 h[c] + mlthf[c] -> nadp[c] + 5mthf[c]	0,169	0,000
MnlDH_rev	mannitol dehydrogenase	nad[c] + mnl[c] <=> h[c] + nadh[c] + man[c]	0,000	0,352
NDPK1	nucleoside-diphosphate kinase (ATP:GDP)	atp[c] + gdp[c] <=> gtp[c] + adp[c]	-6,255	-0,704
NDPK2	nucleoside-diphosphate kinase (ATP:UDP)	atp[c] + udp[c] <=> adp[c] + utp[c]	0,859	0,000
NDPK3	nucleoside-diphosphate kinase (ATP:CDP)	atp[c] + cdp[c] <=> adp[c] + ctp[c]	0,085	0,000
NDPK4	nucleoside-diphosphate kinase (ATP:dTDP)	atp[c] + dtdp[c] <=> adp[c] + dttp[c]	0,001	0,000
NDPK5	nucleoside-diphosphate kinase (ATP:dGDP)	atp[c] + dgdp[c] <=> adp[c] + dgtp[c]	0,001	0,000
NDPK6	nucleoside-diphosphate kinase (ATP:dUDP)	atp[c] + dudp[c] <=> adp[c] + dutp[c]	0,000	0,000
NDPK7	nucleoside-diphosphate kinase (ATP:dCDP)	atp[c] + dcdp[c] <=> adp[c] + dctp[c]	0,001	0,000
NDPK8	nucleoside-diphosphate kinase (ATP:dADP)	atp[c] + dadp[c] <=> adp[c] + datp[c]	0,001	0,000
NGAM2	non growth associated maintenance	1.5 h2o[c] + 1.5 atp[c] -> 1.5 pi[c] + 1.5 h[c] + 1.5 adp[c] + ngam[c]	0,000	0,000
NH4.EQ	Ammonium ammonia equilibrium (cytoplasma)	nh3_[c] + h[c] <=> nh4[c]	-0,815	0,000
NH4.EQ-H	Ammonium equilibrium reaction (chloroplastide)	h[h] + nh3_[h] <=> nh4[h]	0,000	0,000
NO2.RED-H	nitrite reductase (chlorplastides)	7 h[h] + 6 redfer1[h] + no2[h] -> 2 h2o[h] + 6 oxfer[h] + nh3_[h]	0,000	0,000
NO3.RED	nitrate reductase	h[c] + nadh[c] + no3[c] -> h2o[c] + nad[c] + no2[c]	0,000	0,000
NTD11	5'-nucleotidase (IMP)	h2o[c] + imp[c] -> pi[c] + ins[c]	0,188	0,000
NTD7	5'-nucleotidase (AMP)	h2o[c] + amp[c] -> pi[c] + adn[c]	0,000	0,000
NTP1	nucleoside-triphosphatase (ATP)	h2o[c] + atp[c] -> pi[c] + h[c] + adp[c]	0,000	0,000
NTP3	nucleoside-triphosphatase (GTP)	h2o[c] + gtp[c] -> pi[c] + h[c] + gdp[c]	0,000	0,000
NTP5	nucleoside-triphosphatase (CTP)	h2o[c] + ctp[c] -> pi[c] + h[c] + cdp[c]	0,000	0,000

NTPP7	Nucleoside triphosphate pyrophosphorylase (dttp)	h2o[c] + dttp[c] -> ppi[c] + h[c] + dtmp[c]	0,000	0,000
NTPP8	Nucleoside triphosphate pyrophosphorylase (utp)	h2o[c] + utp[c] -> ppi[c] + h[c] + ump[c]	0,000	0,000
OCTRANS.irrev	ornithine carbamoyltransferase	orni[c] + cbp[c] -> pi[c] + h[c] + citr-L[c]	0,186	0,000
OMPDC	orotidine-5'-phosphate decarboxylase	h[c] + orot5p[c] -> co2[c] + ump[c]	0,057	0,000
ORN.AM.TRANS	ornithine aminotransferase	glu-L[c] + glu5sa[c] <=> akg[c] + orni[c]	0,186	0,000
ORPT	orotate phosphoribosyltransferase	ppi[c] + orot5p[c] <=> orot[c] + prpp[c]	-0,057	0,000
OXDHC	2 oxoglutarate Dehydrogenase complex	coa[m] + nad[m] + akg[m] -> nadh[m] + co2[m] + succoa[m]	0,000	0,704
Ox_Phosph(FADH)i-M	overall Oxidative phosphorilation for FADH in mitochondria	15 h[m] + 10 fadh2[m] + 15 adp[m] + 15 pi[m] + 5 o2[m] -> 15 atp[m] + 25 h2o[m] + 10 fad[m]	0,000	0,070
Ox_Phosph(NADH)2i-M	overall Oxidative phosphorilation for NADH in mitochondria	10 nadh[m] + 34 h[m] + 24 adp[m] + 24 pi[m] + 5 o2[m] -> 24 atp[m] + 10 nad[m] + 34 h2o[m]	1,418	0,387
PC-C	pyruvate carboxylase	pyr[c] + atp[c] + hco3[c] -> pi[c] + h[c] + oaa[c] + adp[c]	0,941	0,000
PDH-H	pyruvate dehydrogenase Complex (Chloroplastide)	coa[h] + nad[h] + pyr[h] -> accoa[h] + nadh[h] + co2[h]	0,000	0,000
PDH-M	pyruvate dehydrogenase Complex	coa[m] + nad[m] + pyr[m] -> accoa[m] + nadh[m] + co2[m]	4,337	0,704
PEP.TRANS.P.2	Pohosphoenolpyruvate transphosphorylase	h[c] + pep[c] + gdp[c] -> gtp[c] + pyr[c]	6,292	0,000
PEP.TRANS.P3	phosphoenolpyruvate transphosphorylase	h[c] + pep[c] + dadp[c] -> pyr[c] + datp[c]	0,000	0,000
PEP.TRANS.P4	phosphoenolpyruvate transphosphorylase	h[c] + pep[c] + dgdp[c] -> pyr[c] + dgtp[c]	0,000	0,000
PFK	phosphofructokinase	atp[c] + f6p[c] -> h[c] + adp[c] + fdp[c]	0,000	0,352
PFK-H	phosphofructokinase (Chloroplast)	atp[h] + f6p[h] -> h[h] + adp[h] + fdp[h]	0,000	0,000
PGD	phosphogluconate dehydrogenase (cytoplasma)	nadp[c] + 6pgc[c] -> nadph[c] + co2[c] + ru5p-D[c]	0,000	0,000
PGD-H	phosphogluconate dehydrogenase (Chloroplast)	nadp[h] + 6pgc[h] -> nadph[h] + co2[h] + ru5p-D[h]	0,000	0,000
PGI	glucose-6-phosphate isomerase	g6p[c] <=> f6p[c]	-1,798	0,000
PGI-H	glucose-6-phosphate isomerase	g6p[h] <=> f6p[h]	0,000	0,000
PGK	phosphoglycerate kinase	atp[c] + 3pg[c] <=> adp[c] + 13dpg[c]	-4,098	0,352
PGKrev-H	phosphoglycerate kinase (chloroplast)	atp[h] + 3pg[h] <=> adp[h] + 13dpg[h]	79,826	-1,056
PGL	6-phosphogluconolactonase	h2o[c] + 6pgl[c] -> h[c] + 6pgc[c]	0,000	0,000
PGL-H	6-phosphogluconolactonase (Chloroplast)	h2o[h] + 6pgl[h] -> h[h] + 6pgc[h]	0,000	0,000
PGLYCP-H	Phosphoglycolate phosphatase (Chloroplast)	h2o[h] + 2pglyc[h] -> pi[h] + glyclt[h]	0,091	0,000
PGM	phosphoglycerate mutase	2pg[c] <=> 3pg[c]	-4,098	0,352
PGM-H	phosphoglycerate mutase (chloroplastide)	2pg[h] <=> 3pg[h]	-2,288	-1,056
PGMT	phosphoglucomutase	g1p[c] <=> g6p[c]	-1,795	0,000
PHETA1	phenylalanine transaminase	akg[c] + phe-L[c] <=> glu-L[c] + phpyr[c]	-0,042	0,000
PPCK	phosphoenolpyruvate carboxykinase	oaa[c] + atp[c] -> co2[c] + adp[c] + pep[c]	0,000	0,000
PPI.PHOS	Pyrophosphatase	h2o[c] + ppi[c] -> 2 pi[c] + h[c]	2,185	0,000
PR.ATP.DIPHOS	phosphoribosyl-ATP diphosphatase	h2o[c] + prbatp[c] <=> ppi[c] + h[c] + prbamp[c]	0,001	0,000
PRAGSr.ir	phosphoribosylglycinamide synthase	atp[c] + gly[c] + pram[c] -> pi[c] + h[c] + adp[c] + gar[c]	0,225	0,000
PRAI.C.rev	phosphoribosylaminoimidazole carboxylase (reversible)	co2[c] + air[c] <=> h[c] + 5aizc[c]	0,225	0,000
PRAI.SYNT	phosphoribosylaminoimidazole synthase	atp[c] + fpram[c] -> pi[c] + 2 h[c] + adp[c] + air[c]	0,225	0,000
PRAMP.CYCL	phosphoribosyl-AMP cyclohydrolase	h2o[c] + prbamp[c] <=> prfp[c]	0,001	0,000
PRASCS	phosphoribosylaminoimidazolesuccinocarboxamide synthase	asp-L[c] + atp[c] + 5aizc[c] -> pi[c] + h[c] + adp[c] + 25aics[c]	0,225	0,000
PREDH2	prephenate dehydrogenase	nad[c] + pphn[c] <=> co2[c] + nadh[c] + 34hpp[c]	0,001	0,000
PREDHA	prephenate dehydratase	h[c] + pphn[c] <=> h2o[c] + co2[c] + phpyr[c]	0,042	0,000
PRFGS	phosphoribosylformylglycinamidine synthase	h2o[c] + gln-L[c] + atp[c] + fgam[c] -> pi[c] + h[c] + glu-L[c] + adp[c] + fpram[c]	0,225	0,000

PRKB-H	phosphoribulokinas (Chloroplast)	atp[h] + ru5p-D[h] -> h[h] + adp[h] + r15bp[h]	41,103	0,000
PRMICI	1-(5-phosphoribosyl)-5-[(5-phosphoribosylamino)methylideneamino)imidazole-4-carboxamide isomerase	prfp[c] <=> prlp[c]	0,001	0,000
PROLSYNT3	proline oxidase (prolin biosynthesis)	2 h[c] + nadh[c] + 1pyr5c[c] <=> nad[c] + pro-L[c]	-9999,942	-10000,000
PROLSYNT4	proline oxidase (prolin biosynthesis)	nadph[c] + 2 h[c] + 1pyr5c[c] <=> nadp[c] + pro-L[c]	10000,000	10000,000
PROT.BIOM1.6	Protein Biomass; autotroph Clamyd. Rein	0.067831 asp-L[c] + 0.081155 glu-L[c] + 0.081155 gln-L[c] + 0.27375 ala-L[c] + 0.10296 gly[c] + 0.1502 arg-L[c] + 0.067831 asn-L[c] + 0.0024225 cys-L[c] + 0.020592 ser-L[c] + 0.018169 lys-L[c] + 0.0012113 his-L[c] + 0.032704 ile-L[c] + 0.082366 leu-L[c] + 0.0024225 met-L[c] + 0.033916 phe-L[c] + 0.047239 pro-L[c] + 0.082366 thr-L[c] + 0.0012113 trp-L[c] + 0.0012113 tyr-L[c] + 0.059352 val-L[c] -> 1.2101 h2o[c] + protein.biomass1.6[c]	1,237	0,000
PROT.DEGRADi	Protein Degradation (irreversible. only AS which could be catabolized)	1.2101 h2o[c] + protein.biomass1.6[c] -> 0.0678 asp-L[c] + 0.0812 glu-L[c] + 0.0812 gln-L[c] + 0.2738 ala-L[c] + 0.103 gly[c] + 0.0678 asn-L[c] + 0.0024 cys-L[c] + 0.0206 ser-L[c] + 0.0472 pro-L[c] + 0.0824 thr-L[c] + 0.0012 trp-L[c]	0,000	0,000
PRPP.ATP.TRANS	ATP phosphoribosyltransferase	ppi[c] + prbatp[c] <=> atp[c] + prpp[c]	-0,001	0,000
PRPPS	phosphoribosylpyrophosphate synthetase	atp[c] + r5p[c] <=> h[c] + amp[c] + prpp[c]	0,285	0,000
PYK	pyruvate kinase	h[c] + adp[c] + pep[c] -> pyr[c] + atp[c]	0,000	0,000
PYK-H	pyruvate kinase (chloroplastide)	h[h] + adp[h] + pep[h] -> atp[h] + pyr[h]	0,000	0,704
Photoreduction2	Photoreduction / lightreaction (extracellular to chloroplastide)	h[c] + 2 nadp[h] + 3 adp[h] + 3 pi[h] + 8 hn[h] -> h2o[c] + 3 atp[h] + 2 nadph[h] + o2[h]	48,480	0,000
RBFK	riboflavin kinase	atp[c] + ribflv[c] -> h[c] + adp[c] + fmn[c]	0,000	0,000
RBL.P.TRANS	D-ribulose 5-phosphotransferase	h[c] + adp[c] + ru5p-D[c] <=> atp[c] + rbl-L[c]	0,745	0,000
REDISO	reductoisomerase	nadph[c] + h[c] + oxbutat[c] <=> nadp[c] + 23dhmb[c]	0,175	0,000
RIBFLV.SYNT	riboflavin synthase	2 dmlz[c] -> 4r5au[c] + ribflv[c]	0,001	0,000
RNDR1	ribonucleoside-diphosphate reductase (ADP)	adp[c] + trdrd[c] -> h2o[c] + dadp[c] + trdox[c]	0,001	0,000
RNDR2	ribonucleoside-diphosphate reductase (GDP)	gdp[c] + trdrd[c] -> h2o[c] + dgdp[c] + trdox[c]	0,001	0,000
RNDR3	ribonucleoside-diphosphate reductase (CDP)	cdp[c] + trdrd[c] -> h2o[c] + dcdp[c] + trdox[c]	0,000	0,000
RNDR4	ribonucleoside-diphosphate reductase (UDP)	udp[c] + trdrd[c] -> h2o[c] + dudp[c] + trdox[c]	0,002	0,000
RPE	ribulose 5-phosphate 3-epimerase	ru5p-D[c] <=> xu5p-D[c]	-0,703	0,000
RPE-H	ribulose 5-phosphate 3-epimerase (Chloroplast)	ru5p-D[h] <=> xu5p-D[h]	-27,402	0,000
RPI	ribose-5-phosphate isomerase	r5p[c] <=> ru5p-D[c]	0,043	0,000
RPI-H	ribose-5-phosphate isomerase (Chloroplast)	r5p[h] <=> ru5p-D[h]	13,701	0,000
RubisCO-H	RubisCO Co2 fixation	h2o[h] + co2[h] + r15bp[h] -> 2 h[h] + 2 3pg[h]	41,011	0,000
RubisOX-H	RubisOX Photorespiration	o2[h] + r15bp[h] -> 2 h[h] + 3pg[h] + 2pglyc[h]	0,091	0,000
SATase-M	L-serine acetyltransferase (Mitochondrium)	accoa[m] + ser-L[m] <=> coa[m] + acser[m]	0,006	0,000
SBPASEir	sedoheptulose-bisphosphatase	h2o[c] + s17bp[c] -> pi[c] + s7p[c]	0,000	0,000
SBPASEir-H	sedoheptulose-bisphosphatase (Chloroplast)	h2o[h] + s17bp[h] -> pi[h] + s7p[h]	27,402	0,000
SER.DEAM	serine deaminase	ser-L[c] <=> nh3_[c] + h[c] + pyr[c]	-0,264	0,000
SERSYNir	Serin biosynthesis	h2o[c] + glu-L[c] + nad[c] + 3pg[c] -> pi[c] + h[c] + akg[c] + nadh[c] + ser-L[c]	0,000	0,000
SSAL.DH	succinate-semialdehyde dehydrogenase (NADP)	h2o[c] + nadp[c] + sucsal[c] <=> nadph[c] + 2 h[c] + succ[c]	0,000	0,000
SUCD-M	succinate dehydrogenase (FAD)	fad[m] + succ[m] <=> fadh2[m] + fum[m]	0,000	0,704
SUCOAS-M	succinyl-CoA synthetase (GDP-forming)	coa[m] + succ[m] + gtp[m] <=> pi[m] + succoa[m] + gdp[m]	0,000	-0,704
SUCR.HYLA	sucrose glucohydrolase	h2o[c] + sucr[c] -> fru[c] + glc-D[c]	0,002	0,000
SUCR.SYNT	sucrose synthase	fru[c] + udpg[c] -> h[c] + udp[c] + sucr[c]	0,002	0,000
SULFOX	"	h2o[c] + o2[c] + so3[c] <=> h2o2[c] + so4[c]	-0,023	0,000
SULFRED-M	sulfite reductase (ferredoxin) (Mitochondrium)	8 h[m] + 6 redfer1[m] + so3[m] <=> 3 h2o[m] + h2s[m] + 6 oxfer[m]	-2,992	-0,352
SULFRED-O	sulfite reductase (ferredoxin) (Chloroplastides)	8 h[h] + 6 redfer1[h] + so3[h] <=> 3 h2o[h] + 6 oxfer[h] + h2s[h]	2,998	0,352

TALA	transaldolase	g3p[c] + s7p[c] <=> e4p[c] + f6p[c]	-0,328	0,000
TALA-H	transaldolase (Chloroplast)	g3p[h] + s7p[h] <=> e4p[h] + f6p[h]	13,701	0,000
THRD	L-threonine dehydrogenase	nad[c] + thr-L[c] -> h[c] + nadh[c] + 2aobut[c]	0,102	0,000
THREODEAM	L-threonine deaminase	thr-L[c] <=> 2obut[c] + nh4[c]	0,043	0,000
THRS	threonine synthase	h2o[c] + phom[c] -> pi[c] + thr-L[c]	0,247	0,000
TKT1	transketolase	r5p[c] + xu5p-D[c] <=> g3p[c] + s7p[c]	-0,328	0,000
TKT1-H	transketolase (Chloroplast)	xu5p-D[h] + r5p[h] <=> g3p[h] + s7p[h]	-13,701	0,000
TKT2	transketolase	e4p[c] + xu5p-D[c] <=> f6p[c] + g3p[c]	-0,375	0,000
TKT2-H	transketolase (Chloroplast)	e4p[h] + xu5p-D[h] <=> g3p[h] + f6p[h]	-13,701	0,000
TMD.SYNT	thymidylate synthase	dump[c] + mlthf[c] <=> dhf[c] + dtmp[c]	0,001	0,000
TPI	triose-phosphate isomerase	dhap[c] <=> g3p[c]	-3,320	0,352
TPI-H	triose-phosphate isomerase (Chloroplast)	dhap[h] <=> g3p[h]	-27,402	0,000
TRDR	thioredoxin reductase (NADPH)	nadph[c] + h[c] + trdox[c] -> nadp[c] + trdrd[c]	0,004	0,000
TRPSYNT	Tryptophane synthesis	chor[c] + gln-L[c] + ser-L[c] + prpp[c] <=> h2o[c] + ppi[c] + h[c] + glu-L[c] + pyr[c] + co2[c] + g3p[c] + oh[c] + trp-L[c]	0,001	0,000
TYRTA	tyrosine transaminase	akg[c] + tyr-L[c] <=> glu-L[c] + 34hpp[c]	-0,001	0,000
T_AC_c_h	Acetate transport (cytoplasma to chloroplastides)	ac[c] <=> ac[h]	3,891	0,000
T_AC_c_m	Acetate transport (cytoplasma to mitochondrium)	ac[c] <=> ac[m]	-3,891	0,000
T_ADP_c_h	adp transport (cytoplasma to chloroplast)	adp[c] <=> adp[h]	21,047	1,760
T_ADP_c_m	adp transport (cytoplasma to mitochondrium)	adp[c] <=> adp[m]	34,021	10,352
T_AKG2_c_m	alpha-ketoglutarate (oxo-glutarate) transport (cytoplasma to mitochondrium)	akg[c] <=> akg[m]	-0,446	0,000
T_AMET_c_er	S-Adenosyl-L-methionin Transport (cytoplasma to ER)	amet[c] <=> amet[r]	0,166	0,000
T_AMP_c_h	amp transport (cytoplasma to chloroplast)	amp[c] <=> amp[h]	-4,315	0,000
T_AMP_c_m	amp transport (cytoplasma to mitochondrium)	amp[c] <=> amp[m]	3,885	0,000
T_ANT_CA2-H_c_r	Calcium | Proton Antiporter (extracellular to cytoplasma)	ca2[c] + h[r] <=> h[c] + ca2[r]	23,520	11,760
T_ANT_HCO3-Cl_e_c	Bicarbonate | Chloride antiporter (HCO3 from extracellular to cytoplasma)	hco3[e] + cl[c] <=> hco3[c] + cl[e]	0,000	0,000
T_ATP_c_h	atp transport (cytoplasma to chloroplast)	atp[c] <=> atp[h]	-16,732	-1,760
T_ATP_c_m	atp transport (cytoplasma to mitochondrium)	atp[c] <=> atp[m]	-37,906	-10,352
T_CA2_e_c	Calcium transport (extracellular to cytoplasma)	ca2[e] <=> ca2[c]	23,520	11,760
T_CA2_r_o	Calcium transport via vesicle fusion (ER to Coccolith Vesicles)	ca2[r] <=> ca2[o]	23,520	11,760
T_CMP_c_er	CMP Transport (cytoplasma to ER)	cmp[c] <=> cmp[r]	-0,079	0,000
T_CMP_c_h	CMP transport (cytoplasma to Chloroplastid)	cmp[c] <=> cmp[h]	-0,006	0,000
T_CO2_c_er	CO2 Transport (cytoplasma to ER)	co2[c] <=> co2[r]	-0,070	0,000
T_COA_c_er	COA Transport (cytoplasma to ER)	coa[c] <=> coa[r]	-0,158	0,000
T_COA_c_h	Coenyzme A transport (cytoplasma to chloroplastides)	coa[c] <=> coa[h]	0,158	0,000
T_COA_c_m	Coenzym A transport (cytoplasma to mitochondrium)	coa[c] <=> coa[m]	0,000	0,000
T_CTP_c_er	CTP transport (cytoplasma to ER)	ctp[c] <=> ctp[r]	0,079	0,000
T_CTP_c_h	CTP transport (cytoplasma to Chloroplastid)	ctp[c] <=> ctp[h]	0,006	0,000
T_CYS_c_m	Cysteintransport (extracellular to mitochondria)	cys-L[c] <=> cys-L[m]	-0,006	0,000

T_FA-COA.REPL2_c_m	coa-fatty acid replacement transport (cytoplasma to mitochondrium)	fa-coa.replace_i[c] <=> fa-coa.replace_i[m]	0,000	0,000
T_FA-COA.REPL4_h_c	Transport for fatty acid-coa replacement metabolite (chloroplastide to cytoplasm)	fa-coa.replace_i[h] <=> fa-coa.replace_i[c]	0,158	0,000
T_FA-COA_c_er	fatty acid Coa Transport (cytoplasma to ER)	fa-coa.replace_i[c] <=> fa-coa.replace_i[r]	0,158	0,000
T_FA.REPL4_h_c	Transport for fatty acid replacement metabolite (chloroplastide to cytoplasm)	fa.replace_d[h] <=> fa.replace_d[c]	0,000	0,000
T_FUM_c_m	Fumarate transport (cytoplasma to mitochondrium)	fum[c] <=> fum[m]	0,411	0,000
T_G3P_c_er	Glyceraldehyd 3 Phosphat (cytoplasma to ER)	g3p[c] <=> g3p[r]	0,079	0,000
T_G3P_c_h	Glyceraldehyd 3 Phosphat (cytoplasma to chloroplastide)	g3p[c] <=> g3p[h]	-11,190	1,056
T_GDP_c_m	gdp transport (cytoplasma to mitochondrium)	gdp[c] <=> gdp[m]	0,000	0,704
T_GLN_c_h	Glutamine transport (cytoplasma to chloroplast)	gln-L[c] <=> gln-L[h]	0,000	0,000
T_GLN_c_m	Glutamine transport (cytoplasma to mitochondrion)	gln-L[c] <=> gln-L[m]	0,000	0,000
T_GLU_c_h	Glutamate transport (cytoplasma to chloroplast)	glu-L[c] <=> glu-L[h]	0,000	0,000
T_GLU_c_m	Glutamate transport (cytoplasma to mitochondrion)	glu-L[c] <=> glu-L[m]	0,000	0,000
T_GLYCLT_h_c	Glycolate Transport (Chloroplast to Cytoplasma)	glyclt[h] <=> glyclt[c]	0,091	0,000
T_GLY_c_m	Glycine transport (cytoplasma to mitochondrion)	gly[c] <=> gly[m]	0,012	0,000
T_GTP_c_m	gtp transport (cytoplasma to mitochondrium)	gtp[c] <=> gtp[m]	0,000	-0,704
T_H2O_ch	H2O transport (cytoplasma to Chloroplast)	h2o[c] <=> h2o[h]	55,354	-2,113
T_H2O_cm	H2O transport (cytoplasma to Mitochondrium)	h2o[c] <=> h2o[m]	-38,358	-12,464
T_H2O_e_c	H2O transport (extracellular to cytoplasma)	h2o[e] <=> h2o[c]	50,695	9,295
T_H2S__c_h	Hydrogen sulfide transport (cytoplasma to chloroplast)	h2s[c] <=> h2s[h]	-2,998	-0,352
T_H2S__c_m	Hydrogen sulfide transport (cytoplasma to mitochondrium)	h2s[c] <=> h2s[m]	2,998	0,352
T_HCO3_c_o	Bicarbonate transport (cytoplasma to coccolith vesicle)	hco3[c] <=> hco3[o]	23,520	11,760
T_HCYS_c_er	Homocystein Transport (cytoplasma to ER)	hcys-L[c] <=> hcys-L[r]	-0,166	0,000
T_H_c_h	Proton transport (cytoplasma to chloroplastide)	h[c] <=> h[h]	-24,243	1,408
T_H_c_m	Proton transport (cytoplasma to mitochondrium)	h[c] <=> h[m]	32,375	11,056
T_H_c_o	Proton transport (cytoplasma to coccolith vesicle)	h[o] <=> h[c]	23,520	11,760
T_H_c_r 2	ATPVc/c→ builds up protongradient in ER (H from cytoplasma to ER)	h2o[c] + atp[c] <=> pi[c] + adp[c] + h[r]	23,520	11,760
T_H_e_c	Proton transport (extracellular to cytoplasma)	h[e] <=> h[c]	-49,160	-23,520
T_INOST_c_er	myo-Inositol Transport (cytoplasma to ER)	inost[c] <=> inost[r]	0,003	0,000
T_LIPID-CHLORO_c_h	Chloroplast Lipid transport (cytoplasma to Chloroplastid)	lipid.chloroplast2[c] <=> lipid.chloroplast2[h]	-0,510	0,000
T_LIPID.ER2_c_er	LIPID Transport (cytoplasma to ER)	lipid.er4[r] <=>	0,510	0,000
T_NH4_c_h	Ammonium Transporter (cytoplasma to chloroplastide)	nh4[c] <=> nh4[h]	0,000	0,000

T_NH4_c_m	Ammonium Transporter (cytoplasma to mitochondrion)	nh4[c] <=> nh4[m]	-0,006	0,000
T_NH4_e_c	Ammonium Transporter (extracellular to cytoplasma)	nh4[e] <=> nh4[c]	3,615	0,000
T_O2_c_h	O2 transport (cytoplasma to chloroplastides)	o2[c] <=> o2[h]	-47,176	0,000
T_O2_c_m	O2 transport via diffusion (cytoplasma to mitochondrium)	o2[c] <=> o2[m]	7,088	2,289
T_O2_e_c	O2 transport via diffusion (extracellular to cytoplasma)	o2[e] <=> o2[c]	-40,019	2,289
T_OAA_c_m	Oxalacetate transport (cytoplasma to mitochondrium)	oaa[c] <=> oaa[m]	0,035	0,000
T_PEP_c_h	Phosphoenolpyruvat transport (cytoplasma to chloroplastide)	pep[c] <=> pep[h]	-2,288	-0,352
T_PI_c_er	Phosphat Transport (cytoplasma to ER)	pi[c] <=> pi[r]	-0,012	0,000
T_PPI_c_er	Pyrophosphat Transport (cytoplasma to ER)	ppi[c] <=> ppi[r]	-0,073	0,000
T_PPi_c_h	Pyrophosphat transport (cytoplasma to chloroplastide)	ppi[c] <=> ppi[h]	-4,315	0,000
T_PPi_c_m	Pyrophosphat transport (cytoplasma to mitochondrium)	ppi[c] <=> ppi[m]	3,885	0,000
T_PYR_c_h	pyruvate transport (cytoplasma to chloroplast)	pyr[c] <=> pyr[h]	0,000	-0,704
T_PYR_c_m	pyruvate transport (cytoplasma to mitochondrium)	pyr[c] <=> pyr[m]	4,337	0,704
T_Pi_c_h	Orthophosphat transport (cytoplasma to chloroplastide)	pi[c] <=> pi[h]	34,521	1,056
T_Pi_c_m	Orthophosphat transport (cytoplasma to mitochondrium)	pi[c] <=> pi[m]	34,021	11,056
T_Pi_e_c	Orthophosphat transport (extracellular to cytoplasma)	pi[e] <=> pi[c]	0,669	0,000
T_SER_c_er	Serine Transport (cytoplasma to ER)	ser-L[c] <=> ser-L[r]	0,070	0,000
T_SUCC_c_m	Succinate transport (cytoplasma to mitochondrium)	succ[c] <=> succ[m]	0,000	0,000
T_SYM_HCO3+Na_e_c	Bicarbonate | Sodium Symporter (HCO3 from extracellular to cytoplasma)	na1[e] + hco3[e] <=> hco3[c] + na1[c]	0,000	0,000
T_So3_c_h	Sulfite transport (cytoplasma to chloroplastide)	so3[c] <=> so3[h]	2,998	0,352
T_So3_c_m	Sulfite transport (cytoplasma to mitochondrium)	so3[c] <=> so3[m]	-2,992	-0,352
T_So4_e_c	Sulfat transport (extracellular to cytoplasma)	so4[e] <=> so4[c]	0,023	0,000
T_UDP-GAL_c_h	UDP Galactose transport (cytoplasma to Chloroplastid)	udpgal[c] <=> udpgal[h]	0,136	0,000
T_UDP-SQV_c_h	UDP Sulfoquinovose transport (cytoplasma to Chloroplastid)	udp-sqv[c] <=> udp-sqv[h]	0,017	0,000
T_UDP_c_h	UDP transport (cytoplasma to Chloroplastid)	udp[c] <=> udp[h]	-0,153	0,000
T_hn_e_h	Photon transport (extracellular to chloroplastide)	hn[e] -> hn[h]	387,840	0,000
T_no2_c_h	Nitrite transport (cytoplasma to chloroplastide)	no2[c] <=> no2[h]	0,000	0,000
T_no3_e_c	Nitrate transporter (extracellular to cytoplasma)	no3[e] <=> no3[c]	0,000	0,000
UDP-SQV.SYNT	UDP-sulfoquinovose synthesis	h[c] + udpg[c] + so3[c] <=> h2o[c] + udp-sqv[c]	0,017	0,000
UDPG4E	UDPglucose 4-epimerase	udpg[c] <=> udpgal[c]	1,127	0,000

UDPGALACTAN.SYNT(300)	UDP-Galactan synthesis. UDP-alpha-D-galactose: galactan beta-1.4-D-galactosyltransferase	300 udpgal[c] -> 299 h[c] + udp.galactan(300)[c] + 299 udp[c]	0,000	0,000
UMPK	UMP kinase	atp[c] + ump[c] <=> adp[c] + udp[c]	0,057	0,000
URASE	urease	h2o[c] + urea[c] -> 2 nh3_[c] + co2[c]	0,000	0,000
URIDK2r	uridylate kinase (dUMP)	atp[c] + dump[c] <=> adp[c] + dudp[c]	-0,002	0,000
VALSYNT	Valine Synthetase (branched-chain-amino-acid transaminase)	glu-L[c] + 3mob[c] <=> akg[c] + val-L[c]	0,073	0,000
dTDP.DEPHOS	dTDP nucleotidohydrolase	h2o[c] + dtdp[c] -> pi[c] + h[c] + dtmp[c]	0,000	0,000
dTTP.DEPHOS	dTTP nucleotidohydrolase	h2o[c] + dttp[c] -> pi[c] + h[c] + dtdp[c]	0,000	0,000
t2_mnl[c]_transfer			0,352	0,000
t2_lipid.biomass5.[c]_transfer			0,000	0,000
t2_protein.biomass1.6[c]_transfer			0,000	0,000
t2_lipid.chloroplast2[c]_transfer			0,000	0,000
t2_biomass_log_200_24h_neutrcharge_final[c]_transfer			0,924	0,924
